# Molecular Networking, Network Pharmacology, and Molecular Docking Approaches Employed to Investigate the Changes in Ephedrae Herba before and after Honey-Processing

**DOI:** 10.3390/molecules27134057

**Published:** 2022-06-23

**Authors:** Hengyang Li, Long Guo, Xiaoying Ding, Qi An, Lei Wang, Shenghui Hao, Wenjie Li, Tao Wang, Zetong Gao, Yuguang Zheng, Dan Zhang

**Affiliations:** 1 Traditional Chinese Medicine Processing Technology Innovation Center of Hebei Province, College of Pharmacy, Hebei University of Chinese Medicine, Shijiazhuang 050200, China; lhy56778@163.com (H.L.); guolong@hebcm.edu.cn (L.G.); dingxiaoyingn@163.com (X.D.); 18233106330@163.com (Q.A.); lei.wang@hebcm.edu.cn (L.W.); hshskctbdh@163.com (S.H.); 17370230043@163.com (W.L.); wangtaojywzb@163.com (T.W.); s3060333360@163.com (Z.G.); 2 International Joint Research Center on Resource Utilization and Quality Evaluation of Traditional Chinese Medicine of Hebei Province, Shijiazhuang 050200, China; 3 Department of Pharmaceutical Engineering, Hebei Chemical and Pharmaceutical College, Shijiazhuang 050026, China

**Keywords:** raw Ephedrae herba, honey-processed Ephedrae herba, UPLC-Q-TOF-MS, molecular networking, network pharmacology, molecular docking

## Abstract

Raw Ephedrae herba (REH) and honey-processed Ephedrae herba (HEH) were the different decoction pieces of Ephedrae herba (EH). Honey-processing that changes REH into HEH has been shown to relieve cough and asthma to a synergistic extent. However, the chemical markers and the synergistic mechanism of HEH need to be further studied. In this study, the ultra-high performance liquid chromatography coupled with hybrid quadrupole time of flight mass spectrometry (UPLC-Q-TOF-MS) and molecular networking (MN) were used to investigate the chemical composition of REH and HEH, which led to the identification of 92 compounds. A total of 38 differential chemical markers for REH and HEH were identified using principal component analysis (PCA) and orthogonal partial least squares discriminant analysis (OPLS-DA). Network pharmacology suggests that the synergistic effect of HEH in relieving cough and asthma may be due to 31 differential chemical markers acting through 111 biological targets. Among them, four compounds and two targets probably played an important role based on the results of molecular docking. This study enriched our knowledge about the chemical composition of REH and HEH, as well as the synergistic mechanism of HEH.

## 1. Introduction

According to Leigong Paozhi Lun (a Chinese ancient book), “honey-processing allows drugs become sweet and sluggish with effects of benefiting Qi, moistening lung, relieving cough, and stopping pain and dysentery” [1]. Modern research has shown that honey-processing playing a synergistic role to enhance the therapeutic effect, but also moderating the medicinal properties to reduce side effects, such as honey-processed astragalus [2], honey-processed licorice [3], etc.

Ephedrae herba (EH), deriving from the dried stems of *Ephedra sinica* Stapf, is an herbal plant that commonly grows in northern China [4], which has been widely used in traditional Chinese medicine for the treatment of common cold, coughs, asthma, and edema for thousands of years [5]. The main components of EH include alkaloids, flavonoids, tannins, polysaccharides, organic acids, volatile oils, and many other active compounds [6]. Raw Ephedrae herba (REH) and honey-processed Ephedrae herba (HEH) are the two forms of EH for decoction pieces [7], and HEH has significantly different pharmaceutical properties than REH, as do their clinical applications. This diversification might be due to complex chemical changes taking place during processing.

Liquid chromatography-tandem mass spectrometry (LC-MS) is increasingly used for the rapid identification of the differential components about Chinese medicine processing. However, unequivocal identification by LC-MS is achieved only when reference compounds are available, and we need a more efficient tool to ident these compounds. In recent years, a new technology called Global Natural Products Social Molecular Networking (GNPS) has been developed to characterize natural products and make compound discovery more efficient [8]. Molecular networking (MN) can allow visualization of all molecular ions detected in LC-MS/MS experiments and the chemical relationships between these molecular ions, which plays an important role in rapid identification of compounds and discovery of new compounds [9,10]. Network pharmacology was regarded as an important strategy for the development of TCM by using the method of “multi-component, multi-target, multi-pathway” and combining systems biology, bioinformatics, and pharmacology. Network pharmacology can systematically identify the mechanism of action of multi-component and multi-target TCM, which has been applied to a variety of TCM research [11,12]. Molecular docking is an established method based on a computer simulation structure, which helps to predict the interaction between molecules and biological targets. Through molecular docking, we can verify the binding affinity between active compounds and key targets and improve the accuracy of the target network [13,14].

In this study, UPLC-Q-TOF-MS combined with MN was applied to identify the chemical compounds, and the differential compounds for REH and HEH were found by principal component analysis (PCA) and orthogonal partial least squares discriminant analysis (OPLS-DA). The network pharmacology was used to investigate the synergistic effect of HEH, as well as the multi-component synergistic mechanism, which was further verified by molecular docking.

## 2. Results

### 2.1. HPLC-Q-TOF MS Data and MN Analysis

#### 2.1.1. The Analysis of P1

**Figure 1:**
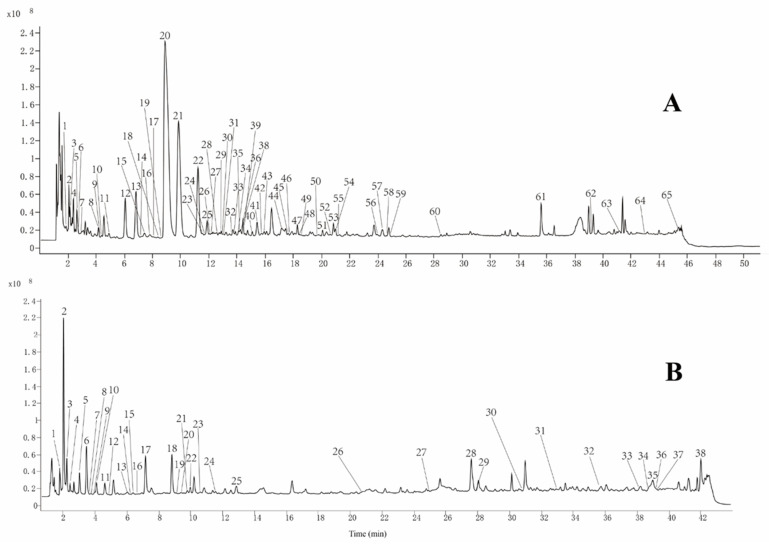
Total ion flow diagram of REH and HEH methanolic extracts obtained by HPLC-Q-TOF MS in positive ion mode. (**A**) fraction P1; (**B**) fraction P2.

**Figure 2:**
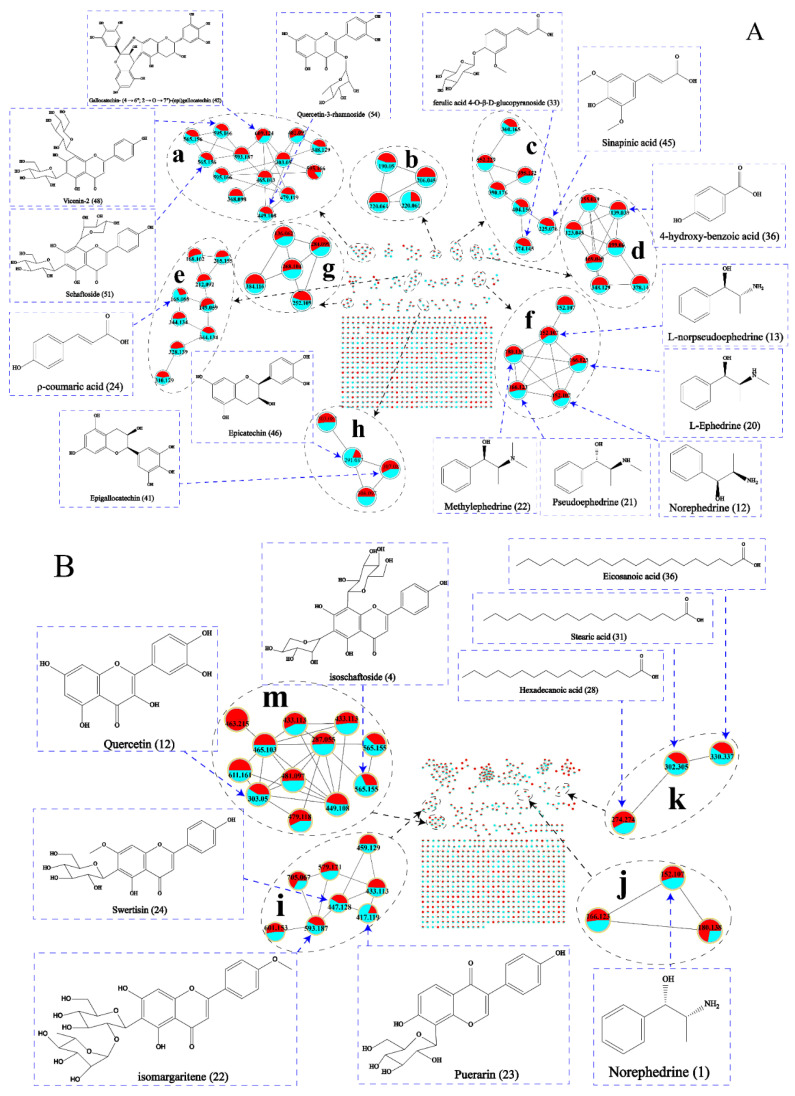
The molecular network of raw Ephedrae herba and honey-processed Ephedrae herba (**A**): fraction P1; (**B**) fraction P2). MN for the nodes annotated as “flavonoid and flavonoid glycosides” class (a, h, i, and m). MN for the nodes annotated as “quinolinic acids” class (b). MN for the nodes annotated as “phenolic acids” class (c, d, and e). MN for the nodes annotated as “alkaloids” class (f, g, j and k). The pie chart for nodes was filled with different colors, red (raw Ephedrae herba) and blue (honey-processed Ephedrae herba), based on the proportion of the intensity of the ion peak corresponding to each metabolite in the two decoction pieces.

Figure 1A shows the total ion flow diagram for fraction P1, and Table 1 shows the identified compounds. The MN was constructed based on MS/MS similar spectra for visualization as shown in Figure 2A. The constructed MN showed a total of 734 precursor ions visualized as nodes in the molecular map, which include 27 groups (node > 2) and 517 unique nodes. The proportion of the red and blue sectors at each node in MN represents the relative content of the compound in REH and HEH. All the compounds were determined from MS data combined with the MN chemical composition database and published literature. According to MS/MS fragment breakage patterns and MN derivation, 65 components were identified in eight major groups (**groups a–h**). All the structures are shown in Figure S1.

##### Identification of Flavonoids and Flavonoid Glycosides

**Groups a** and **h** are flavonoid derivatives, four aglycones including quercetin (peak 59, *m*/*z* 303.0504, [M + H]^+^), hesperetin/homoeriodictyol (peak 32, *m*/*z* 303.0867, [M + H]^+^), aromadendrin (peak 38, *m*/*z* 306.0973, [M + NH_4_]^+^), and tricin (peak 14, *m*/*z* 348.1291, [M + NH_4_]^+^). In each type of flavonoid structure, the common substituents on the A and B rings include hydroxyl, methyl, and methoxy. The loss of these neutral fragments is the basic fracture pathway of flavonoids. Most of the flavonoid glycosides were *O*-glycosides, a feature evident from the neutral loss of rhamnopyranosyl (*m*/*z* 146) and glucose residues (*m*/*z* 162). The MS/MS fragments of [M-90]^+^ and [M-120]^+^ were produced by typical C-glycoside breaks, suggesting that both glycosyl groups are linked to the flavonoid glycosides as C-glycoside bonds, including schaftoside (peak 51, *m*/*z* 565.1556, [M + H]^+^), isoschaftoside (peak 53, *m*/*z* 565.1556, [M + H]^+^), and vicenin-2 (peak 48, *m*/*z* 595.1661, [M + H]^+^).

##### Identification of Quinolinic Acids

**Group b** is quinoline, including 6-methoxykynurenic acid (peak 44, *m*/*z* 220.0609, [M + H]^+^), kynurenic acid (peak 31, *m*/*z* 190.0497, [M + H]^+^), and 6-hydroxykynurenic acid (peak 19, *m*/*z* 206.0451, [M + H]^+^).

##### Identification of Phenolic Acids

**Groups c**, **d,** and **e** are the derivatives of phenolic acids. We identified two components: sinapinic acid (peak 45, *m*/*z* 225.0760, [M + H]^+^) and 1-*O*-β-d-glucopyranosyl sinapate (peak 40, *m*/*z* 404.1558, [M + NH_4_]^+^) by MN in **group c** that has not been reported in the EH-related literature. Another fragment produced during the cleavage of peak 45 (*m*/*z* 225.0760) is at *m*/*z* 178, which is caused by the first cleavage of the methyl group in the precursor ions [15]. In MN, peak 40 (*m*/*z* 404.1558) showed correlation as a node connected with sinapinic acid (peak 45) and syringin (peak 39). 1-*O*-β-d-glucopyranosyl sinapate first loses [C_6_H_11_O_5_]^+^ and [NH_4_]^+^ to produce a fragment ion at *m*/*z* 225 and then loses [H_2_O] to produce a major fragment ion at *m*/*z* 207 [16]. The aglycones of **group d** are vanillic acid (peak 18, *m*/*z* 169.0495, [M + H]^+^), syringic acid (peak 26, *m*/*z* 199.0601, [M + H]^+^) and benzoic acid (peak 28, *m*/*z* 123.0445, [M + H]^+^), and **group e** are cinnamic acid (peak 43, *m*/*z* 149.0596, [M + H]^+^) and ferulic acid (peak 5, *m*/*z* 212.0923, [M + NH_4_]^+^). In addition, with the help of MN, we inferred a glycoside derivative ferulic acid 4-*O*-β-d-glucopyranoside (peak 33, *m*/*z* 374.1446, [M + NH_4_]^+^) related to ferulic acid (peak 5) that has not been reported in the EH-related literature.

##### Identification of Alkaloids

**Groups f** and **g** represent alkaloid derivatives. **Group f** consists mostly of ephedrine derivatives, including l-ephedrine (peak 20, *m*/*z* 166.1229, [M + H]^+^), pseudoephedrine (peak 21, *m*/*z* 166.1229, [M + H]^+^), methylephedrine (peak 22, *m*/*z* 180.1384, [M + H]^+^), norephedrine (peak 12, *m*/*z* 152.1070, [M + H]^+^), and l-norpseudoephedrine (peak 13, *m*/*z* 152.1070, [M + H]^+^). The cleavage of ephedrine derivatives generally involves the loss of [H_2_O], followed by the loss of [CH_3_]^+^, and finally the removal of the [NH_2_]^−^. Taking l-ephedrine as an example, the [M-H_2_O + H]^+^ ion at *m*/*z* 148.1120, the further loss of [CH_3_]^+^ to make acquired of an ion at *m*/*z* 133.0886, respectively, the [M-H_2_O-CH_3_-NH_2_ + H]^+^ ion at *m*/*z* 117.0698. **Group g** belongs to the purine alkaloids, which are cordycepin (peak 7, *m*/*z* 252.1092, [M + H]^+^), adenine (peak 1, *m*/*z* 136.0621, [M + H]^+^), adenosine (peak 4, *m*/*z* 268.1045, [M + H]^+^), and isoguanosine (peak 6, *m*/*z* 284.0992, [M + H]^+^). Adenosine (peak 4) loss of [C_4_H_3_N_4_] or ribosome (*m*/*z* 132) to make acquired of the ion at *m*/*z* 161.0431 or *m*/*z* 136.0619. Isoguanosine and cordycepin are not reported in EH-related literature. Peak 6 loses a moiety of *m*/*z* 132 and then shows a molecular ion peak at *m*/*z* 152.0566, which is similar to adenosine. In MN, peak 6 as a node linking to cordycepin, adenosine, and adenine shows structural correlation and is 16 Da different from adenosine. Therefore, it is determined that peak 6 is isoguanosine.

##### Others

Three other compounds were also first identified from EH, including glucosyringic acid (peak 27, *m*/*z* 378.1339, [M + NH_4_]^+^), gallocatechin-(4 → 6″; 2 → O → 7″)-(epi)gallocatechin (peak 42, *m*/*z* 609.1241, [M + H]^+^), and 3-[2-(4-Hydroxy-3-methoxyphenyl)-3-[[2-*O*-(beta-d-glucopyranosyl)-alpha-d-glucopyranosyloxy]methyl]-7-methoxy-2,3-dihydrobenzofuran-5-yl]-1-propanol (peak 52, *m*/*z* 702.2963, [M + NH_4_]^+^).

#### 2.1.2. The Analysis of Fraction P2

The total ion flow diagram of the fraction P2 is shown in Figure 1B, and the identified chemical compositions are shown in Table 2. The constructed MN (Figure 2B) was shown a total of 742 precursor ions visualized as nodes in the molecular map, which include 26 groups (node > 2) and 456 unique nodes. At each node in MN, the proportion of red and blue sectors represents the relative content of the compound in REH and HEH. The MS data along with the MN chemical composition database and published literature were used to determine these compounds. A total of 38 components including four major groups (**group i**–**m**) were identified based on the MS/MS fragment fracture pattern and MN derivation. The structures of all 38 compounds are shown in Figure S2.

##### Identification of Flavonoids

**Groups i** and **m** are flavonoids, and the main aglycones include kaempferol (peak 20, *m*/*z* 287.0551, [M + H]^+^) and quercetin (peak 12, *m*/*z* 303.0504, [M + H]^+^). Myricetin-3-Galactosid(peak 5, *m*/*z* 481.0976, [M + H]^+^) has not been reported in the literature with EH. In MN, peak 5 as a node linking to kaempferol, hyperoside (peak 11), and quercetin shows structural correlation. Peak 5 has a 16 Da difference with hyperoside and a 178 Da difference with quercetin. Peak 5 produced molecular ion peaks of *m*/*z* 319.0443 [M + H-C_6_H_11_O_5_]^+^ and *m*/*z* 273.0312 [M + H-C_6_H_10_O_5_-CO-H_2_O]^+^, respectively, which are the same as those reported in the literature. Therefore, peak 5 was identified as myricetin-3-galactosid.

##### Identification of Fatty Acids and Alkaloids

**Group k** is fatty acid compounds including stearic acid (peak 31, *m*/*z* 302.3053, [M + NH_4_]^+^), hexadecanoic acid (peak 28, *m*/*z* 274.2741, [M + NH_4_]^+^), and eicosanoic acid (peak 36, *m*/*z* 330.3370, [M + NH_4_]^+^). **Group j** consists mostly of ephedrine derivatives, including norephedrine (peak 1, *m*/*z* 152.1070, [M + H]^+^), l-ephedrine/pseudoephedrine (peak 2, *m*/*z* 166.1230, [M + H]^+^), and methylephedrine (peak 3, *m*/*z* 180.1384, [M + H]^+^).

##### Others

Five others have not been reported in the literature related to EH, including secoisolariciresinol (peak 19, *m*/*z* 345.1590, [M-H_2_O + H]^+^), 7b,9-Dihydroxy-3-(hydroxymethyl)-1,1,6,8-tetramethyl-5-oxo-1,1a,1b,4,4a,5,7a,7b,8,9-decahydro-9aH-cyclopropa[3,4]benzo[1,2-e]azulen-9a-yl acetate (peak 30, *m*/*z* 432.2378, [M + CAN + H]^+^), byzantionoside B (peak 17, *m*/*z* 373.2220, [M + H]^+^), 2-(Hydroxymethyl)-6-[5-[3-(hydroxymethyl)-5-(3-hydroxypropyl)-7-methoxy-2,3-dihydro-1-benzofuran-2-yl]-2-methoxyphenoxy]oxane-3,4,5-triol (peak 13, *m*/*z* 540.2434, [M + NH_4_]^+^), and Griseofulvin (peak 27, *m*/*z* 353.0788, [M + H]^+^).

Based on the MN, 92 components were identified by the MN method, and the structure and detailed mass spectral information of some of the markers are shown in Figure 2, Table 1 and Table 2, respectively. Figure 2A shows that the red sectors of the alkaloid composition for **group f** are larger than the blue sectors. This indicates that the content of alkaloids, the main components of EH, decreases slightly after processing. In **group a**, vicenin-2, schaftoside, kaempferol-3-glucoside-7-rhamnoside, and quercetin-3-rhamnoside have smaller proportions in the red sectors than in the blue, indicating an increase in their levels after processing. At the same time, the red sectors of gallocatechin- (4 → 6″; 2 → O → 7″)-(epi) gallocatechin is greater than the blue, indicating that the content of this component decreases after processing.

### 2.2. PCA and OPLS-DA Analyses for the Identified of Discriminatory Metabolites

**Figure 3:**
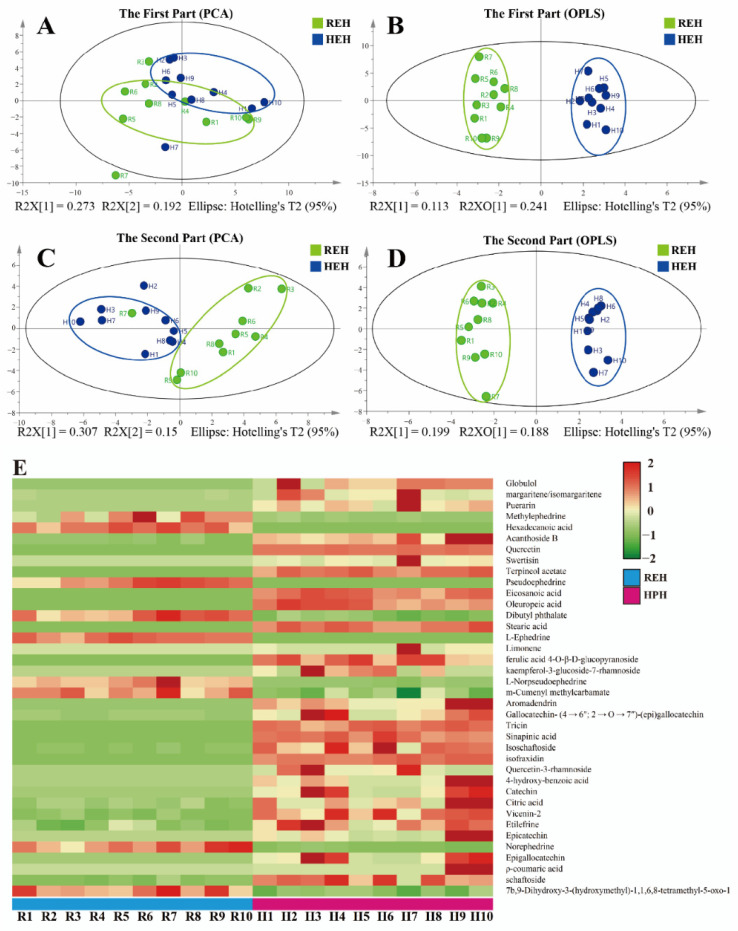
MVA plots of REH and HEH based on the MS spectral data generated in the positive mode. the PCA score plot with 95% density ellipses (**A**,**C**); the OPLS-DA score plot with 95% density ellipses (**B**,**D**); the heatmap of differential compounds related to REH and HEH (**E**).

To verify the accuracy of the MN data analysis, a comparative analysis of the REH and HEH data files was performed. The PCA score spots of REH and HEH were shown in Figure 3A,C. OPLS-DA analysis was shown in Figure 3B,D, indicating significant differences in the components of REH and HEH. Furthermore, the screening of differentially abundant metabolites in REH and HEH was performed based on the variable importance in projection value (VIP > 1) and *p*-value (*p* < 0.05); then, 38 differential chemical markers were screened. Heatmap analysis of these differential chemical markers (Figure 3E) showed that 7b, 9-Dihydroxy-3-(hydroxymethyl)-1,1,6,8-tetramethyl-5-oxo-1,1a,1b,4,4a,5,7a,7b,8,9-decahydro-9aH-cyclopropa[3,4]benzo[1,2-e]azulen-9a-yl acetate, norephedrine, m-cumenyl methylcarbamate, l-norpseudoephedrine, l-ephedrine, dibutyl phthalate, pseudoephedrine, hexaxdecanoic acid and methylephedrine decreased after honey-processing, while other components increased, which is consistent with the results obtained previously by MN. This may be significantly correlated with the synergistic effect of HEH.

### 2.3. Network Pharmacology Analysis

#### 2.3.1. Prediction of the Target Proteins

**Figure 4:**
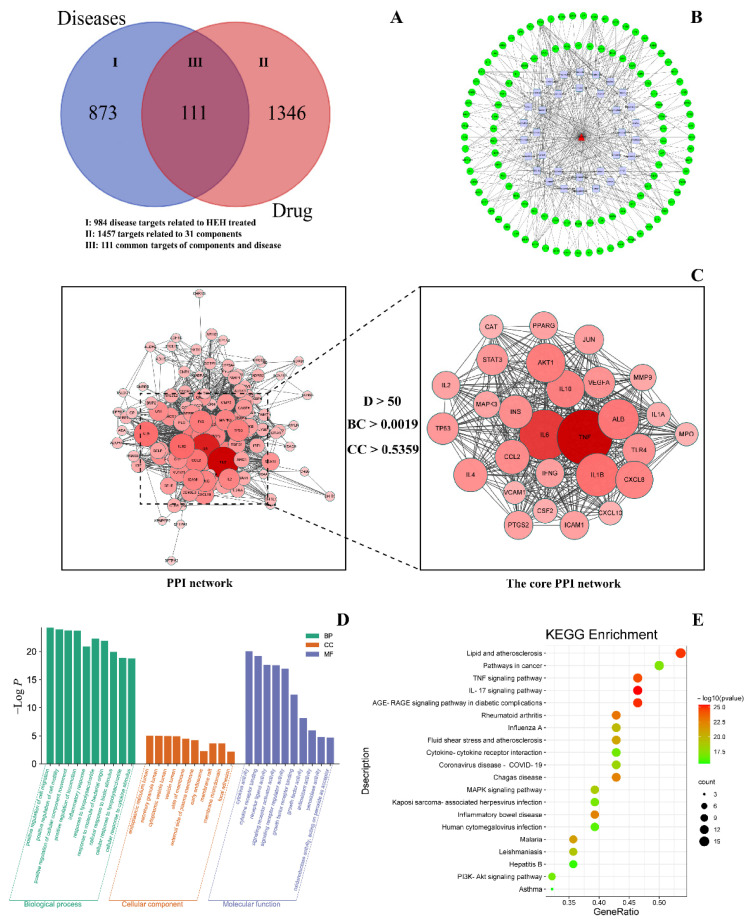
(**A**) Venn diagram of differential chemical markers and intersection targets; (**B**) component-disease-target network; (**C**) PPI network; (**D**) secondary classification histogram of GO functional enrichment analysis. The abscissa shows the GO term, and the ordinate shows the number of genes enriched in the GO term; (**E**) bubble diagram of the KEGG pathway enrichment analysis results. The abscissa shows the ratio of the number of targets genes belonging to a pathway to the total number of annotated genes located in the pathway. The ordinate shows the pathway term. The bubble size indicates the number of targets in the pathway. The bubble color represents the magnitude of the *p*-value. The redder the color, the smaller the *p*-value, and the higher is the degree of enrichment.

In total, 1457 targets related to 31 components of the 38 differential chemical markers and 984 targets associated with diseases treated by HEH were identified. The 111 overlapping component and disease targets were obtained by Venn diagrams (Figure 4A) that is intersecting the component targets and the disease targets.

#### 2.3.2. Construction of the Component-Disease-Target Network

A component-disease-target network was developed to identify the relationships between the 31 active components and 111 overlapping component and disease targets, and Cytoscape V3.8.2 (La Jolla, CA, USA) was used for network visualization and topological analysis. The component-disease-target network contained 143 nodes and 394 edges (Figure 4B).

Degree, Closeness Centrality, and Betweenness Centrality are index of network node centrality. The median value of the Degree (D) was 6, the median value of Closeness Centrality (CC) was 0.3559, and the median value of Betweenness Centrality (BC) was 0.0012. The hub nodes of degree ≥ 2 D with Closeness Centrality ≥ CC and Betweenness Centrality ≥ BC were selected as the core active components (Table 3). These hub nodes included Quercetin (degree = 52), Citric acid (degree = 35), Puerarin (degree = 20), Epicatechin (degree = 15), l-ephedrine (degree = 15), Pseudoephedrine (degree = 13), norephedrine (degree = 12), and tricin (degree = 12) (Table 3). These components might be the core components of EH in the treatment of disease.

#### 2.3.3. Construction of the PPI Network

The 111 overlapping component and disease targets, which were considered the therapeutic targets of HEH in the treatment of cough and asthma, were imported into the STRING database, and the PPI network was constructed. The hub nodes of degree ≥ 2 D with Closeness Centrality ≥ CC and Betweenness Centrality ≥ BC were selected as the core PPI network (Figure 4C). The sizes and colors of the nodes are proportional to the degree. The larger the node and the darker the color, the stronger is the interaction, indicating that the interaction plays a more central role in the PPI network. In total, 28 core targets and 376 edges representing protein–protein interactions were predicted. The top 10 core targets were TNF (degree = 85), IL6 (degree = 84), IL1B (degree = 79), ALB (degree = 76), AKT1 (degree = 74), IL10 (degree = 72), CXCL8 (degree = 71), CCL2 (degree = 67), INS (degree = 66), and IL4 (degree = 66) (Table 3).

#### 2.3.4. GO and KEGG Enrichment Analyses

The 111 overlapping components and disease targets were imported to OmicShare tools for GO functional enrichment and KEGG pathway enrichment analyses. In total, 833 GO terms with *p* < 0.05, including biological processes, cellular components, and molecular functions, were obtained. From these terms, the functional information and secondary classification histogram were drawn with OmicShare tools (Figure 4D). The highest correlations in GO enrichment were associated with cell motility, movement of intracellular components, and inflammation, including positive regulation of cell migration, positive regulation of cell motility, positive regulation of cellular component movement, and positive regulation of locomotion. In total, 132 KEGG pathway items with *p* < 0.05 were obtained, and we selected the 20 top-ranking pathways based on their *p*-values and drew bubble plots with OmicShare tools (Figure 4E).

The largest number of KEGG-enriched targets was Lipid and atherosclerosis, which included 15 targets. Cough and asthma treatment with EH was primarily associated with anti-inflammatory activity, and the enriched signaling pathways for TNF, MAPK, and PI3K-Akt were associated with inflammation. The PI3K-Akt pathway and asthma were associated with the main synergistic effects of HEH on cough and asthma [17,18], with the PI3K-Akt signaling pathway being enriched to a total of 10 targets. PI3K is a member of the lipid kinase family, and Akt is a downstream actor of PI3K. The PI3K-Akt pathway is involved in a variety of roles in vivo, such as antioxidant [19], anti-inflammatory [20], etc. MAPK signaling pathway is also a pathway of anti-inflammatory mechanism in vivo [21].

In summary, these differential chemical markers act on multiple targets and pathways to exert their pharmacological effects, and the variation in the levels of the differential chemical markers differentiates the efficacy of HEH from that of REH. GO and KEGG analyses suggest that HEH may have therapeutic and inflammatory effects as well as antiviral and antioxidant effects. This study provides more ideas for the pharmacological study of HEH.

### 2.4. Component-Target Molecular Docking

**Figure 5:**
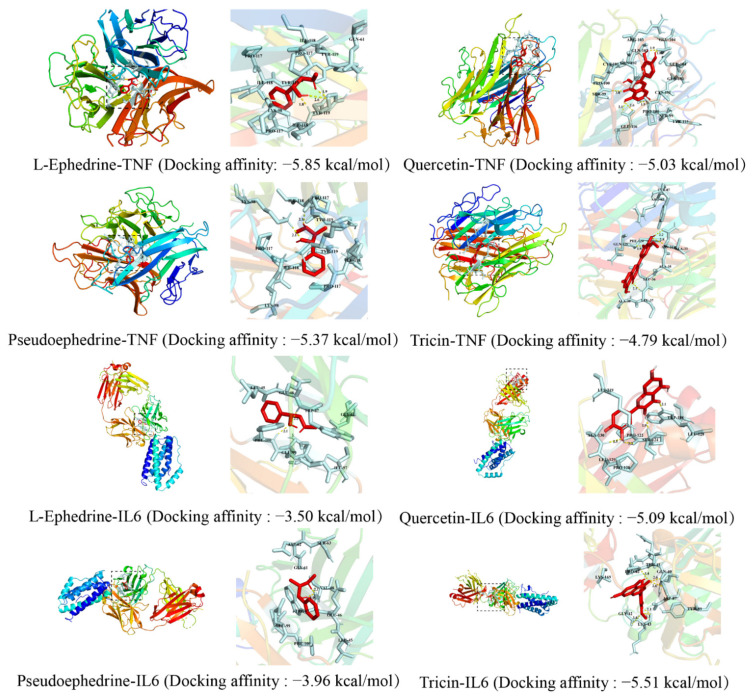
Molecular docking of active ingredients and bub targets.

l-Ephedrine and pseudoephedrine, the main components of EH, quercetin and tricin, whose content increases after honey-processing, were selected to dock with the core target proteins TNF and IL6. The above compounds acting on these target proteins may exert anti-inflammatory and anti-cancer effects through biological pathways such as apoptosis and metabolic cell death. It is generally accepted that the more negative the docking affinity, the more likely it is to bind, and the result of the docking affinity is recorded in Figure 5.

The results show that all four compounds have the potential to bind to all two targets, and that the binding of components and amino acid residues is mainly through hydrogen bonds and van der Waals forces. The molecular docking results further validate that HEH may play an important role in the pathways screened out by network pharmacology and provide data to support further studies of HEH.

## 3. Materials and Methods

### 3.1. Plant Material and Chemicals

LC-MS grade methanol, acetonitrile, and formic acid were purchased from Fisher Scientific (Pittsburgh, PA, USA). Ultrapure water was prepared by a Synergy water purification system (Millipore, Billerica, MA, USA). The internal standard of hyperoside (≥98.0%, Lot no. Y20A9X59340) was purchased from Chengdu Push-Biotechnology Co. Ltd. (Chengdu, China). Other chemicals and reagents were of analytical grade. Ephedrae herba was acquired across the major pharmacies and identified as *Ephedra sinica* Stapf by Dr. Dan Zhang. The details of each sample are listed in Table 4. The specimens were stored at Hebei University of Chinese Medicine.

### 3.2. Sample Preparation for UPLC-Q-TOF-MS Analysis

An aliquot of 1.00 g of sample powder was immersed in 20 mL of 65% methanol (*v*/*v*), followed by ultrasonic extraction at room temperature for 30 min. The mixture was then centrifuged at 13,000 rpm for 10 min, and 1 mL of the extracts was separated into fraction P1 and P2 using a semi-preparative HPLC (Shimadzu Co., Kyoto, Japan). P1 was then diluted 60-fold in 65% methanol and P2 20-fold, 0.00125 mg/mL of hyperoside as an internal standard.

### 3.3. UPLC-Q-TOF-MS Analysis

The UPLC-MS analysis was performed on an Agilent 1290 Infinity II system coupled with an Agilent 6545 quadrupole time-of-flight mass spectrometer system (Q-TOF-MS) (Agilent Technologies, Santa Clara, CA, USA) equipped with an electrospray ionization interface.

A Waters Acquity UPLC HSS T3 column (2.1 × 100 mm, 1.8 μm) was used for sample separation with the flow rate of 0.3 mL/min and at the column temperature of 35 °C. The binary gradient elution system consisted of acetonitrile (B) and water containing 0.1% formic acid (A). The gradient elution of P1 was optimized as follows: 0–8 min, 5% B; 8–10 min, 5–10% B; 10–26 min, 10–20% B; 26–29 min, 20–30% B; 29–34 min, 30–40% B; 34–37 min, 40–50% B; 37–40min, 50–60% B; 40–43 min, 60–70% B. The gradient elution of P2 as: 0–3 min, 20% B; 3–20 min, 20–35% B; 20–27 min, 35–50% B; 27–32min, 50–60% B; 32–36 min, 60–70% B; 36–40 min, 70–82% B. The injection volume was set to 1.0 µL.

The MS acquisition parameters were as follows: drying gas (N2) temperature, 320 °C; sheath gas temperature, 350 °C; drying gas (N2) flow rate, 10.0 L/min; sheath gas flow (N2) rate, 11 L/min; nebulizer gas pressure, 35 psi; capillary voltage, 3500 V; fragmentor voltage, 135 V; collision energy, 40 eV. The analysis was operated in a negative mode with the mass range of *m*/*z* 100–1000 Da. Data were analyzed by MassHunter Qualitative Analysis Software Version B.10.00 (Agilent Technologies, Palo Alto, CA, USA). Quality control (QC) samples were prepared by pooling the same amount of samples together, and every 5th was utilized as a QC sample.

### 3.4. Establishment of Molecular Networking

The MN was constructed using the UPLC-Q-TOF-MS/MS data from REH and HEH. All MS/MS data files were converted into 32-bit mzXML by using MSconvert software. The converted files were uploaded to the GNPS platform (https://gnps.ucsd.edu, 25 March 2022) via WinSCP (https://winscp.net, 25 March 2022), following the online workflow to build the MN: https://ccms-ucsd.github.io/GNPSDocumentation/quickstart (25 March 2022). The created MN and parameters can be accessed via the link: http://gnps.ucsd.edu/ProteoSAFe/status.jsp?task=3902109bbf36453ca44f4fa5a553c85b (25 March 2022). The MN parameters were as follows: minimum cosine score 0.70; minimum matched peaks 6; tolerance 0.02 Da for parent mass and fragments; maximum connected component size 100; minimum cluster size 1, no run MScluster. The results were exported to Cytoscape 3.8.2 software for visualization.

### 3.5. Mass Spectrometry Analysis and Metabolites Annotation

The LC-MS data acquisition was conducted on a MassHunter Workstation (Agilent Technologies). After normalization, the data set was introduced into SIMCA software (version 13.0, Umetrics, Umea, Sweden), for PCA and OPLS-DA. Heatmap analysis was generated by OriginPro 2019b software (Origin Lab Corporation, Northampton, MA, USA).

### 3.6. Network Pharmacology

To reveal the correlation between the differential compounds and the synergistic effect of relieving cough and asthma of HEH, and to predict the potential targets and pathways closely associated with the synergistic of HEH from a comprehensive perspective, a network pharmacology study was performed in this study.

#### 3.6.1. Construction of Networks

Firstly, the differential compounds between REH and HEH were uploaded to the Traditional Chinese Medicine Systems Pharmacology Database and Analysis Platform (TCMSP, https://old.tcmsp-e.com/tcmsp.php, 9 April 2022), Integrative Pharmacology-based Research Platform of Traditional Chinese Medicine (TCMIP, http://www.tcmip.cn/TCMIP/index.php, 9 April 2022), HERB (http://drug.ac.cn, 9 April 2022), BATMAN-TCM (http://bionet.ncpsb.org.cn/batman-tcm/index.php/Home/Index/index, 9 April 2022), and SwissTarget-Prediction database (http://swisstargetprediction.ch, 9 April 2022) to obtain the compound-related targets.

Secondly, with relieving asthma, asthma, cough, relieving cough, anti-inflammatory, inflammatory and antioxidant as the keywords, the related targets were collected using the following database: GeneCards (https://www.genecards.org, 10 April 2022), Online Mendelian Inheritance in Man (OMIM, https://www.omim.org, 10 April 2022), Therapeutic Target Database (TTD, http://db.idrblab.net/ttd, 10 April 2022), TCMSP, the Encyclopedia of Traditional Chinese Medicine (ETCM, http://www.tcmip.cn/ETCM/index.php/Home/Index, 10 April 2022), and SymMap (http://www.symmap.org, 10 April 2022). After the collected targets were merged and removed duplication, the core targets were obtained by intersecting the targets of differential compounds and diseases based on the STRING database (https://cn.string-db.org, 10 April 2022).

Thirdly, each core target (enzymes, receptors, transporters, cytokines, proteins, and others) was classified to construct networks of differential compounds-core targets and the protein interaction (PPI). The network was generated by Cytoscape software (version 3.8.2) (http://www.cytoscape.org, 12 April 2022) with topological analysis. Through the topological analysis, the main differential compounds and the potential targets were screened out.

#### 3.6.2. Kyoto Encyclopedia of Genes and Genomes (KEGG) and Gene Ontology (GO) Pathway Enrichment Analysis

The targets were input to the Database for Annotation, Visualization, and Integrated Discovery (DAVID) (https://david.ncifcrf.gov/summary.jsp, 13 April 2022) for Gene Ontology (GO) and Kyoto Encyclopedia of Genes and Genomes (KEGG) pathway analysis, and species were selected as “homo sapiens”. OmicShare’s platform tool (http://www.omicshare.com/tools, 13 April 2022) was used to draw the bubble plot. The color of the dot represents different *p*-values, and the size of the dot reflects the number of target genes expressed in the pathway. The rich factor represents the ratio of all target genes in a pathway to the number of all the annotated genes in the pathway. A higher rich factor represents a higher level of enrichment.

### 3.7. Component-Target Molecular Docking

AutoDock Tools (vision 4.2.6, La Jolla, CA, USA) is a receptor–ligand docking simulation program used for protein–ligand docking simulation and prediction of the docking affinity [22]. Molecular docking analysis was adopted to confirm the interactions between the core components and the core targets of REH and HEH in the treatment of asthma and to verify the accuracy of the network pharmacology prediction. The three-dimensional (3D) structures of the target proteins were downloaded from the PDB database (https://www.rcsb.org/, 18 April 2022), and the MOL2 structures of the differential compounds were downloaded from the TCMSP database. We removed the water molecules, separated the proteins, added nonpolar hydrogens, calculated the Gasteiger charges for each structure, and saved the data as a PDBQT file. The target proteins were receptors, and the active components were ligands. AutoDock Vina 1.1.2 (La Jolla, CA, USA) was used to dock molecules with proteins. The Vina score is the core of the complex obtained by docking the receptor and ligand with the corresponding pocket parameters using the Vina procedure. The lower the Vina score is, the higher the affinity of the receptor and ligand. It is generally believed that, when the conformation of the ligand and receptor is stable, the lower the energy and the greater the possibility of interaction. When the docking affinity is less than 0, the ligand and the receptor can bind spontaneously. The conformation with the best affinity was selected as the final docking conformation and visualized with PyMOL 2.3 (New York, NY, USA).

## 4. Conclusions

In this study, UPC-Q-TOF-MS combined with the MN were used to identify the components of REH and HEH for the first time, and 92 compounds were tentatively identified, including 27 flavonoids, 9 alkaloids, 26 phenolic acids, 3 quinolinic acids, 6 fatty acids, and 21 others, and 38 differential chemical markers were screened out by MN and OPLS-DA. The synergistic mechanism of HEH was studied using network pharmacology and molecular docking. Thirty-one active components were probably acting through 111 biological targets, and the enriched signaling pathways for TNF, MAPK, and PI3K-Akt were associated with cough and asthma. Among them, four components and two targets probably played an important role. This study enriched our knowledge about the chemical composition and the synergistic mechanism of HEH.

## Figures and Tables

**Table 1:** Information on 65 compounds in P1 identified by HPLC-Q-TOF-MS.

No.	*t_R_* (min)	Molecular Formula	Quasi-Molecular	MS/MS Fragments	Error (ppm)	Identification
1	1.88	C_5_H_5_N_5_	[M + H]^+^ 136.0621	109.0499; 119.0349; 136.0621	2.43	Adenine
2&	2.06	C_6_H_8_O_7_	[M + NH_4_]^+^ 210.0612	193.1207; 175.0588; 113.9623	1.94	Citric acid
3	2.16	C_15_H_18_O_8_	[M + H]^+^ 344.1342	327.1263; 344.0911; 165.0544; 147.0455; 119.0492	0.6	*O*-Coumaric acid glucoside
4	2.41	C_10_H_13_N_5_O_4_	[M + H]^+^ 268.1045	161.0431; 136.0619; 119.0350	1.76	Adenosine
5	2.68	C_10_H_10_O_4_	[M + NH_4_]^+^ 212.0923	212.0922; 194.0809; 152.0706; 134.0599	2.91	Ferulic Acid
6	2.68	C_10_H_13_N_5_O_5_	[M + H]^+^ 284.0992	152.0566	0.9	Isoguanosine
7	2.69	C_10_H_13_N_5_O_3_	[M + H]^+^ 252.1092	136.0619	0.34	Cordycepin
8	4.14	C_17_H_24_O_7_	[M + H]^+^ 358.1864	340.1748; 179.2667; 162.7284	1.04	Nebrodenside A
9&	4.16	C_10_H_15_NO_2_	[M + H]^+^ 182.1178	182.1178; 164.1069	1.35	Etilefrine
10	4.34	C_15_H_18_O_7_	[M + NH_4_]^+^ 328.1393	311.1288; 132.0810	0.71	4-β-d-glucopyranosyloxy-trans-Cinnamaldehyde
11	4.94	C_9_H_10_O_2_	[M + NH_4_]^+^ 168.1021	105.0702; 133.0647	1.3	4-Vinylguaiacol
12&	6.07	C_9_H_13_NO	[M + H]^+^ 152.107	152.1118; 134.0964; 117.0695	0.06	Norephedrine
13&	6.83	C_9_H_13_NO	[M + H]^+^ 152.107	152.1118; 134.0964; 117.0695	0.06	l-norpseudoephedrine
14&	7.20	C_17_H_14_O_7_	[M + NH_4_]^+^ 348.1291	331.1776; 314.2340	−2.06	Tricin
15	7.40	C_15_H_14_O_7_	[M + H]^+^ 307.0815	223.0600; 205.0504; 195.0645; 181.0474; 177.0554; 169.0510; 139.0388; 127.0389	0.88	Gallocatechin
16	7.74	C_7_H_6_O_4_	[M + H]^+^ 155.0338	140.0594; 138.0553; 137.0229; 111.0439; 110.0604; 109.0280	−0.55	Protocatechuic acid
17	8.05	C_14_H_18_O_9_	[M + NH_4_]^+^ 348.1287	169.0491	−0.6	Pseudolaroside B
18	8.07	C_8_H_8_O_4_	[M + H]^+^ 169.0495	152.0703; 112.9556; 110.0086	−0.21	Vanillic acid
19	8.75	C_10_H_7_NO_4_	[M + H]^+^ 206.0451	178.0467; 162.0442; 160.0394	1.54	6-hydroxykynurenic acid
20&	9.04	C_10_H_15_NO	[M + H]^+^ 166.1229	148.1120; 133.0886; 117.0698	1.57	l-ephedrine
21&	9.91	C_10_H_15_NO	[M + H]^+^ 166.1229	148.1120; 133.0886; 117.0698	1.57	Pseudoephedrine
22&	11.25	C_11_H_17_NO	[M + H]^+^ 180.1384	180.1384; 162.1277; 147.1047; 117.0701	0.61	Methylephedrine
23	11.53	C_14_H_20_N_2_O_3_	[M + H]^+^ 265.1548	265.1520; 177.0544; 145.0279; 117.0322	0.5	(*trans*)*cis*-*N*-feruloylputrescine
24	11.55	C_9_H_8_O_3_	[M + H]^+^ 165.0547	137.1092; 124.0850; 121.0637; 115.0537; 102.0356	0.48	ρ-coumaric acid
25	11.87	C_13_H_18_N_4_O_3_	[M + H]^+^ 279.1454	279.1456; 220.0974; 105.0332	0.84	N″-benzoyl-l-arginine
26&	12.03	C_9_H_10_O_5_	[M + H]^+^ 199.0601	182.0524; 165.0140; 140.0472; 125.0958	0	Syringic acid
27	12.03	C_15_H_20_O_10_	[M + NH_4_]^+^ 378.1399	199.0594; 155.0703	1.19	Glucosyringic acid
28	12.58	C_7_H_6_O_2_	[M + H]^+^ 123.0445	123.0427; 108.0618; 106.0732	3.64	Benzoic acid
29	12.87	C_23_H_34_O_14_	[M + NH_4_]^+^ 552.2287	193.0859; 161.0595	0.03	Isosyringinoside
30	12.88	C_11_H_12_O_3_	[M + H]^+^ 193.0861	161.0602; 136.0738	0.93	Myristicin
31	12.95	C_10_H_7_NO_3_	[M + H]^+^ 190.0497	162.0318; 144.0440	−0.9	Kynurenic acid
32	13.66	C_16_H_14_O_6_	[M + H]^+^ 303.0867	151.0384; 153.0558; 137.0602; 119.0493; 123.0434	1.28	Hesperetin/Homoeriodictyol
33&	13.71	C_16_H_20_O_9_	[M + NH_4_]^+^ 374.1446	195.0648; 151.0743; 177.0549	0.12	Ferulic acid 4-*O*-β-d-glucopyranoside
34	14.10	C_9_H_8_O_3_	[M + H]^+^ 165.0547	121.0745; 119.0740; 117.0691	0.48	*o*-coumaric acid
35&	14.10	C_15_H_18_O_8_	[M + NH_4_]^+^ 344.1342	344.0911; 326.1246; 165.0544; 149.0785; 119.0492	0.6	ρ-coumaric acid glucoside
36&	14.21	C_7_H_6_O_3_	[M + H]^+^ 139.0397	139.0389; 122.0957; 111.0442	5.28	4-hydroxy-benzoic acid
37&	14.23	C_15_H_14_O_6_	[M + H]^+^ 291.0867	291.0870; 165.0539; 139.0392	1.33	Catechin
38&	14.23	C_15_H_12_O_6_	[M + NH_4_]^+^ 306.0973	289.0693; 261.1972; 179.0330; 153.0532	0.3	Aromadendrin
39	14.41	C_17_H_24_O_9_	[M + NH_4_]^+^ 390.1764	211.0956	1.46	Syringin
40	15.06	C_17_H_22_O_10_	[M + NH_4_]^+^ 404.1558	225.0758; 207.0653; 192.0405	1.75	1-*O*-β-d-glucopyranosyl sinapate
41&	15.36	C_15_H_14_O_7_	[M + H]^+^ 307.0815	223.0600; 205.0504; 195.0645; 177.0554; 169.0510; 139.0388;	0.88	Epigallocatechin
42&	15.75	C_30_H_24_O_14_	[M + H]^+^ 609.1241	441.0809; 303.0499	0.36	Gallocatechin- (4 → 6″; 2 → O → 7″)-(epi)gallocatechin
43	15.93	C_9_H_8_O_2_	[M + H]^+^ 149.0596	104.0579; 131.0496	−0.72	Cinnamic acid
44	17.14	C_11_H_9_NO_4_	[M + H]^+^ 220.0609	220.0598; 202.0485; 174.0553; 146.0598	2.13	6-methoxykynurenic acid
45	17.63	C_11_H_12_O_5_	[M + H]^+^ 225.076	225.0761; 181.0817; 151.0377	1.12	Sinapinic acid
46&	17.66	C_15_H_14_O_6_	[M + H]^+^ 291.0867	134.0966; 117.0697; 115.0542; 106.0655	1.33	Epicatechin
47&	18.28	C_10_H_16_	[M + H]^+^ 137.1326	137.0626; 121.0406; 107.0847	0.9	Limonene
48&	18.31	C_27_H_30_O_15_	[M + H]^+^ 595.1661	577.1549; 457.1129; 379.0810; 337.0708	0.59	Vicenin-2
49	18.48	C_8_H_8_O_3_	[M + H]^+^ 153.0546	109.0647; 135.0433; 125.0953	−0.14	4-hydroxyphenylacetic acid
50&	19.77	C_27_H_30_O_15_	[M + H]^+^ 595.1661	287.0552; 449.1089; 329.0275;	0.59	kaempferol-3-glucoside-7-rhamnoside
51&	20.09	C_26_H_28_O_14_	[M + H]^+^ 565.1556	565.1516; 475.1224; 445.1148; 355.0911;	0.74	Schaftoside
52	20.67	C_32_H_44_O_16_	[M + NH_4_]^+^ 702.2963	331.1540; 343.1572;	1.74	3-[2-(4-Hydroxy-3-methoxyphenyl)-3-[[2-*O*-(beta-d-glucopyranosyl)-alpha-d-glucopyranosyloxy] methyl]-7-methoxy-2,3-dihydrobenzofuran-5-yl]-1-propanol
53&	20.87	C_26_H_28_O_14_	[M + H]^+^ 565.1556	565.1516; 475.1224; 445.1148; 355.0911	0.74	Isoschaftoside
54&	21.02	C_21_H_20_O_11_	[M + H]^+^ 449.1082	299.0555; 109.0273; 153.0572; 229.0473; 272.9334	0.81	Quercetin-3-rhamnoside
55	21.17	C_27_H_30_O_15_	[M + H]^+^ 595.1661	287.0552; 449.1089; 329.0275	0.59	Kaempferol-3-*O*-rhamnoside 7-Oglucoside
56	23.94	C_10_H_8_O_4_	[M + H]^+^ 193.0497	193.0507; 178.0266; 149.0239; 133.0293; 121.0658	0.86	Scopoletin
57	24.40	C_26_H_34_O_11_	[M + NH_4_]^+^ 540.2447	493.1487; 239.1038	1.46	Urolignoside
58	24.79	C_21_H_20_O_12_	[M + H]^+^ 465.1034	303.0503; 257.0440; 153.0187	1.4	Hyperoside
59&	24.79	C_15_H_10_O_7_	[M + H]^+^ 303.0504	257.0445; 229.0494; 285.0392; 303.0501	1.56	Quercetin
60	28.55	C_22_H_22_O_12_	[M + H]^+^ 479.1196	363.2410; 317.0653; 302.0433; 255.0016; 245.0436	0.41	Isorhamnetin-3-*O*-glucoside
61&	35.94	C_12_H_20_O_2_	[M + NH_4_]^+^ 214.1806	136.1056	2.27	Terpineol acetate
62&	39.17	C_16_H_32_O_2_	[M + NH_4_]^+^ 274.2741	274.2738; 257.2679; 226.1808; 212.2357; 197.0965	0.17	Hexadecanoic acid
63&	41.15	C_11_H_15_NO_2_	[M + H]^+^ 194.1175	137.0566; 194.1180	−0.29	m-Cumenyl methylcarbamate
64&	42.97	C_11_H_10_O_5_	[M + H]^+^ 223.0603	223.0609; 208.0361; 163.0394	0.9	Isofraxidin
65	45.71	C_22_H_43_NO	[M + H]^+^ 338.3419	149.1355; 212.2030; 303.3041; 338.3424	0.47	Erucylamide

**Table 2:** Information on 38 compounds in P2 identified by HPLC-Q-TOF-MS.

No.	*t_R_* (min)	Molecular Formula	Quasi-Molecular	MS/MS Fragments	Error (ppm)	Identification
1&	1.822	C_9_H_13_NO	[M + H]^+^ 152.107	152.1118; 134.0964; 117.0695	0.06	Norephedrine
2&	2.022	C_10_H_15_NO	[M + H]^+^ 166.123	166.1230; 148.1121; 133.0887; 117.0697	2.18	l-ephedrine/Pseudoephedrine
3&	2.222	C_11_H_17_NO	[M + H]^+^ 180.1384	180.1384; 162.1277; 147.1047; 117.0701	0.61	Methylephedrine
4&	2.521	C_26_H_28_O_14_	[M + H]^+^ 565.1552	565.1552; 475.1048; 445.1132; 355.0797	0.03	Isoschaftoside/schaftoside
5	2.971	C_21_H_20_O_13_	[M + H]^+^ 481.0976	319.0443; 273.0312	−0.14	Myricetin-3-Galactoside
6	3.337	C_27_H_30_O_14_	[M + H]^+^ 579.1711	579.2605; 433.1123; 283.0597	0.46	Vitexin-2-rhamnoside
7	3.354	C_21_H_20_O_10_	[M + H]^+^ 433.1133	433.1151; 271.0591	0.87	Apigenin-7-*O*-glucoside
8	3.637	C_27_H_30_O_16_	[M + H]^+^ 611.1607	611.2231; 465.1023; 303.0502	0.06	Rutin
9&	3.853	C_10_H_16_	[M + H]^+^ 137.1325	107.0846; 121.0391; 137.1087	0.17	Limonene
10	3.970	C_21_H_20_O_10_	[M + H]^+^ 433.1133	433.1149; 415.1036; 313.0710; 283.0604; 397.0921; 379.0809; 337.0714	0.87	Vitexin/Isovitexin
11	4.452	C_21_H_20_O_12_	[M + H]^+^ 465.1031	303.0501; 257.0445; 137.0235; 153.0178	0.75	Hyperoside
12&	4.452	C_15_H_10_O_7_	[M + H]^+^ 303.0504	257.0445; 229.0494; 285.0392; 303.0501	1.56	Quercetin
13	6.001	C_26_H_34_O_11_	[M + NH_4_]^+^ 540.2434	331.1534; 313.1419; 287.1281; 151.0755; 137.0573	−1.03	2-(Hydroxymethyl)-6-[5-[3-(hydroxymethyl)-5-(3-hydroxypropyl)-7-methoxy-2,3-dihydro-1-benzofuran-2-yl]-2-methoxyphenoxy]oxane-3,4,5-triol
14	6.217	C_9_H_8_O_2_	[M + H]^+^ 149.0597	149.0597; 105.0352	−0.04	Cinnamic acid/Trans-cinnamic acid
15&	6.578	C_28_H_36_O_13_	[M + NH_4_]^+^ 598.2492	401.1590; 205.0859	−0.37	Acanthoside B
16&	6.700	C_21_H_20_O_11_	[M + H]^+^ 449.1082	299.0555; 109.0273; 153.0572; 229.0473; 272.9334	0.81	Quercetin-3-rhamnoside
17	7.100	C_19_H_32_O_7_	[M + H]^+^ 373.222	135.1167; 175.1474; 193.1586; 211.1688	−0.21	Byzantionoside B
18	8.831	C_11_H_12_O_4_	[M + H]^+^ 209.0809	177.0544; 191.0697; 121.0643	0.31	Sinapaldehyde
19	9.597	C_20_H_26_O_6_	[M-H_2_O + H]^+^ 327.1590	137.0599; 163.0747; 133.0647; 138.0630	−0.26	Secoisolariciresinol
20	9.547	C_15_H_10_O_6_	[M + H]^+^ 287.0551	153.0184; 287.0575; 213.0544; 165.0159	0.3	Kaempferol
21	9.564	C_21_H_20_O_10_	[M + H]^+^ 433.1133	287.0554; 286.0484	0.87	Kaempferol-3-rhamnoside
22&	9.714	C_28_H_32_O_14_	[M + H]^+^ 593.1868	593.1819; 473.1448; 447.1285; 429.1186; 285.0742	0.54	Margaritene/isomargaritene
23&	10.396	C_21_H_20_O_9_	[M + H]^+^ 417.1192	417.1225; 399.1037; 381.0957	2.86	Puerarin
24&	11.528	C_22_H_22_O_10_	[M + H]^+^ 447.1285	297.0758; 327.0871; 447.1264	−0.16	Swertisin
25	12.610	C_16_H_28_O_6_	[M + H]^+^ 317.1957	137.1327	−0.52	(−)-α-terpineol-8-*O*-β-d-glucopyranoside
26&	20.452	C_12_H_20_O_2_	[M + NH_4_]^+^ 214.1803	136.1207	0.74	Terpineol acetate
27	24.881	C_17_H_17_ClO_6_	[M + H]^+^ 353.0788	165.0544; 215.0100; 285.0529; 353.0781	0.45	Griseofulvin
28	27.511	C_16_H_32_O_2_	[M + H]^+^274.2741	196.9640; 257.2673; 274.2741	0.16	Hexadecanoic acid
29&	27.911	C_15_H_26_O	[M + NH_4_]^+^ 240.2323	107.0848; 109.1002	0.49	Globulol
30&	30.741	C_22_H_30_O_6_	[M + CAN + H]^+^ 432.2378	119.0853; 135.0799; 147.0643; 281.1375; 107.0855	-	7b,9-Dihydroxy-3-(hydroxymethyl)-1,1,6,8-tetramethyl-5-oxo-1,1a,1b,4,4a,5,7a,7b,8,9-decahydro-9aH-cyclopropa[3,4]benzo[1,2-e]azulen-9a-yl acetate
31&	32.922	C_18_H_36_O_2_	[M + NH_4_]^+^ 302.3053	302.3055; 285.2978; 241.2370	−0.2	Stearic acid
32	35.869	C_18_H_30_O_2_	[M + H]^+^ 279.2319	237.9922; 109.101	0.16	Linolenic acid
33&	38.000	C_10_H_16_O_3_	[M + H]^+^ 185.1172	111.0443	−0.66	Oleuropeic acid
34	38.500	C_18_H_32_O_2_	[M + NH_4_]^+^ 298.2742	298.2742; 281.1532; 263.2369; 245.2253; 239.2349	0.51	9,12-Linoleic acid
35&	38.833	C_16_H_22_O_4_	[M + H]^+^ 279.1594	149.0232; 150.0268; 223.9774; 279.1594	1.13	Dibutyl phthalate
36&	38.833	C_20_H_40_O_2_	[M + NH_4_]^+^ 330.337	257.1545; 227.1744; 330.3365; 312.3233	1.1	Eicosanoic acid
37	39.066	C_18_H_34_O_3_	[M + H]^+^ 321.2425	119.0856; 151.1119; 133.1012; 161.0931	0.25	(*E*)-6-hydroxyoctadec-4-enoic acid
38	42.046	C_22_H_43_NO	[M + H]^+^ 338.3418	338.3410; 303.3043; 212.2006; 149.1316	0.17	Erucylamide

Note: & Represent for differential markers.

**Table 3:** The 8 core components and 10 core targets of HEH in disease treatment.

Classification	No.	Name	Degree	Closeness Centrality	Betweenness Centrality
Core components	1	Quercetin	52	0.4814	0.1050
	2	Citric acid	35	0.4316	0.0524
	3	Puerarin	20	0.3767	0.0120
	4	Epicatechin	15	0.3827	0.0091
	5	l-Ephedrine	15	0.3807	0.0101
	6	Pseudoephedrine	13	0.3747	0.0055
	7	Norephedrine	12	0.3559	0.0050
	8	Tricin	12	0.3727	0.0071
Core targets	1	TNF	85	0.7943	0.0543
	2	IL6	84	0.7832	0.0588
	3	IL1B	79	0.7568	0.0459
	4	ALB	76	0.7467	0.0570
	5	AKT1	74	0.7368	0.0468
	6	IL10	72	0.7226	0.0267
	7	CXCL8	71	0.7179	0.0195
	8	CCL2	67	0.6957	0.0151
	9	INS	66	0.6957	0.0199
	10	IL4	66	0.6914	0.0300

**Table 4:** Information of Raw Ephedrae Herba (REH) samples used in this study.

Sample	Botanical Origin	Collection Area
REH-1	Ephedra sinica Stapf	Hebei Hou Xiaobin Chinese Medicine Clinic
REH-2		Hebei Tian Chunji Chinese Medicine Clinic
REH-3		Hebei Zhizi Pharmacy on Hongqi Street
REH-4		Hebei Bai xingkang Pharmacy
REH-5		Hebei Tongrentang pharmacy
REH-6		Hebei Emerging pharmacy
REH-7		Hebei Shenwei Pharmacy
REH-8		Hebei Lerentang Pharmacy
REH-9		Hebei Anguo Market
REH-10		Hebei Guoyitang Pharmacy

## Data Availability

Not applicable.

## Supplementary Materials

The following supporting information can be downloaded at: https://www.mdpi.com/article/10.3390/molecules27134057/s1, Table S1. Regression equations and linear ranges of 3 compounds; Table S2. Response surface experimental design factor and level code; Table S3. Responses for the Box-Behnken experimental design; Table S4. ANOVA for response surface models; Figure S1. Structures of 65 compounds in P1 identified by HPLC-Q-TOF-MS; Figure S2. Structures of 38 compounds in P2 identified by HPLC-Q-TOF-MS; Figure S3. 3D view of response surface methodology of honey-processing Ephedrae herba. (A) Mutual effects of Processing time and Processing temperature on OD value; (B) Mutual effects of Processing time and Infiltration time on OD value; (C) Mutual effects of Processing temperature and Infiltration time on OD value.

## Sample Availability

Not available.
