# Supplementary material for: Molecular Networking, Network Pharmacology, and Molecular Docking Approaches Employed to Investigate the Changes in Ephedrae Herba before and after Honey-Processing
Source: Molecules. 2022 Jun 23;27(13):4057. doi: 10.3390/molecules27134057 (PMC9268138; doi:10.3390/molecules27134057)

Supplementary Materials

Molecular Networking, Network Pharmacology, and Molecular Docking Approaches Employed to Investigate the Changes in Ephedrae Herba before and after Honey-Processing

Hengyang Li [1,†], Long Guo [1,2,†], Xiaoying Ding [1], Qi An [1], Lei Wang [1,2], Shenghui Hao [1], Wenjie Li [1], Tao Wang [1], Zetong Gao [1], Yuguang Zheng [1,3,*] and Dan Zhang [1,2,*]

[1] Traditional Chinese Medicine Processing Technology Innovation Center of Hebei Province, College of Pharmacy, Hebei University of Chinese Medicine, Shijiazhuang 050200, China; lhy56778@163.com (H.L.); guolong@hebcm.edu.cn (L.G.); dingxiaoyingn@163.com (X.D.); 18233106330@163.com (Q.A.); lei.wang@hebcm.edu.cn (L.W.); hshskctbdh@163.com (S.H.); 17370230043@163.com (W.L.); wangtaojywzb@163.com (T.W.); s3060333360@163.com (Z.G.)

[2] International Joint Research Center on Resource Utilization and Quality Evaluation of Traditional Chinese Medicine of Hebei Province, Shijiazhuang 050200, China

[3] Department of Pharmaceutical Engineering, Hebei Chemical and Pharmaceutical College, Shijiazhuang 050026, China

* Correspondence: zyg314@163.com (Y.Z.); zhangdan@hebcm.edu.cn (D.Z.); Tel.: +86-311-8511-0166 (Y.Z.); +86-311-8992-6466 (D.Z.)

† These authors contributed equally to this paper.

Table S1. Regression equations and linear ranges of 3 compounds.

Table S2. Response surface experimental design factor and level code.

Table S3. Responses for the Box–Behnken experimental design.

Table S4. ANOVA for response surface models.

Figure S1. Structures of 65 compounds in P1 identified by HPLC-Q-TOF-MS.

Figure S2. Structures of 38 compounds in P2 identified by HPLC-Q-TOF-MS.

Figure S3. 3D view of response surface methodology of honey-processing Ehphedrae herba. (A) Mutual effects of Processing time and Processing temperature on OD value; (B) Mutual effects of Processing time and Infiltration time on OD value; (C) Mutual effects of Processing temperature and Infiltration time on OD value.

1. Sample Preparation

1.1. Sample Preparation of Total Alkaloids in Ephedrae herba

An aliquot of 0.60 g of sample powder was immersed in 26 mL of 0.5 % hydrochloric acid (V/V), followed by ultrasonic extraction at room temperature for 30 min. The mixture was then centrifuged at 13,000 rpm for 10 min, and the supernatant was diluted to 60 mg/mL with 0.5 % hydrochloric acid. In addition, 1 mL of the diluted solution should be mixed with 4 mL acetic acid-ammonium acetate buffer solution (pH 4.5) and 4 mL 0.05 % bromocresol green (dissolved in buffer solution, pH 4.5), followed by 10 mL chloroform, vertexing and shaking for 2 min. The mixture was then centrifuged at 3800 rpm for 10 min. The chloroform layer was transferred to a quartz cuvette, scanned at 416 nm, and the results were recorded.

1.2. Sample Preparation of Total Flavonoid in Ephedrae herba

One gram of sample powder was immersed in 25 mL of 65 % methanol (V/V) and then extracted by ultrasonic at room temperature for 12 min. After centrifuging for 10 minutes at 13,000 rpm, the mixture was diluted to 200 mg/mL with 65% methanol. In addition, 0.4 mL of the diluted solution was added to a 5 mL volumetric flask with 1 mL water and 0.2 mL 5% $NaNO_2$ solution, shaken well, and left to stand for 6 min. Then, 0.2 mL 10% solution of $Al(NO_3)_3$ is added, shaken well, and left to stand for 6 min. Finally, 2 mL 4% NaOH solution was added, fixed capacity with water, shaken well, and allowed to stand for 15 min. The mixture was transferred to Multimode Microplate Reader (PerkinElmer Victor Nivo, City, MA, USA), scanned at 506 nm, and the results were recorded.

1.3. Sample Preparation of Total Phenolic Acid in Ephedrae herba

One gram of the sample powder was immersed in 25 ml of 65% methanol (V/V) and extracted for 12 minutes with sonication at room temperature. Following centrifugation at 13,000 rpm for 10 minutes, the mixture was diluted to 200 mg/ml with 65% methanol. In addition, 0.2 ml of the dilution was added to a 5 ml volumetric flask with 2.2 ml of water and 0.2 ml of forint reagent, shaken well, and left for five minutes protected from light. Finally, a 5% of Na_2CO_3 solution was added to fix the volume, shaken well, and left for 40 minutes protected from light. The mixture was transferred to a multimode microplate reader (PerkinElmer Victor Nivo, City, MA, USA), scanned at 711 nm, and the results were recorded.

2. Experimental Design of RSM for the Processing Technology

The total score of total alkaloid content (TAC), total flavonoid content (TFC), and total phenolic acid content (TPAC) were normalized as evaluation indexes to optimize the best processing technology of HEH. Firstly, a single factor experimental design was used to analyze various processing parameters of HEH, including infiltration time at 5-9 h, processing time at 10–14 min, and processing temperature at 90–110 °C were optimized. The normalization is done as follows:

$$d_{n=}(d_i - d_{min}) \div (d_{max} - d_{min})$$

where d_i, d_{min}, and d_{max} are the actual values measured in the experiment. d_{min} and d_{max} are the minimum and maximum values of the set of values, respectively. d_n is the normalized value of d_i. The normalized value of each index was calculated according to the formula to calculate the geometric mean to obtain the overall evaluation OD value.

It is calculated as follows:

$$OD = (d_1\ d_2\ d_3 \cdots d_n)^{1/n}$$

where n is the number of indicators [17].

Design-Expert software (Version 11; Stat-Ease Inc., Minneapolis, MN, USA) was used to optimize the honey-processing of EH. The Box–Behnken Design (BBD) model was used [18, 19]. Based on the results of single-factor experiments, a total of 17 trials were performed based on the design. Table S2 depicts the rank of the independent variables, levels, and experimental design in coded and decoded terms. The OD value was set as the response of the design experiments (Y). The experimental orders, levels of variables, and response values are summarized in Table S3. Data on three independent variables and one response variable were analyzed to get a second-order polynomial model as follows:

$$Y = b_0 + \sum_{i=1}^{3} b_i X_i + \sum\nolimits_{i=1}^{3} b_{ii} X_{ij} + \sum\nolimits_{i<1}^{3} b_{ij} X_i X_j$$

where b_0, b_i, b_{ii}, and b_{ij} $(i \neq j)$ are the regression coefficients for intercept, linear, quadratic, and interaction terms, respectively, and Xi and Xj are the independent variables [20]. The quality of fit of the polynomial model was evaluated for the coefficient of determination (R^2) and F-test. The lack of fit F-value ($p < 0.05$) was acquired by analysis of variance (ANOVA) and used to demonstrate variable significance and model adequacy. To verify the prediction ability and sufficiency of the model, the optimal processing conditions for prediction were verified by experiments.

3. Optimization of Processing Technology of HRE

3.1. Methodological Validation for the Determination of TAC, TFC, and TPAC

To determine the TAC, TFC, and TPAC, the linearity, precision, repeatability, stability, and accuracy of the established method were evaluated. As shown in Table S4, all calibration curves have good linearity with high correlation coefficients ($r^2 \geq 0.9990$) over the tested range. The relative standard deviations (RSD) of intra- and inter-day precisions were less than 0.60%, 0.62%, 0.34%, 1.60%, 0.92%, and 1.54%, respectively. The repeatability, expressed as RSD, was less than 4.93%, 1.62%, and 2.17%, and the stability was less than 0.93%, 1.37%, and 0.21%, respectively. The overall recoveries of the three indicators were between 95.22–100.90%, and the RSD was less than 2.92%. The results of method validation showed that the established method was suitable for the analysis of HRE samples.

3.2. Single Factor Experiment

The purpose of a univariate experiment is to assess the effect of each factor on the OD value and to analyze the effect of three different variables. As shown in Figure 1, when the infiltration time was increased from 5 h to 9 h, the OD value increased from 0 to 0.8302 with time and then decreased with time. When processing time from 10 min to 12 min, the OD value increased significantly, and from 12 min to 14 min, it started to decrease. When the processing temperature is between 90 °C and 110 °C, the maximum OD value is at 100 °C. For this reason, 12 minutes of processing time, 100 °C of processing temperature, and 7 h of infiltration time were selected as the best single factor conditions, and the response surface methodology was further optimized.

3.3. RSM Modeling

The regression ANOVA was performed on the results of Table 1, and the quadratic

polynomial regression equation were obtained for the OD value (Y) on processing time (A), processing temperature (B), and infiltration time (C) Y = 0.74+0.043A-0.065B+0.043C-0.041AB-0.11AC-0.074BC-0.37A^2-0.26B^2-0.084C^2. By performing the ANOVA significance test on the regression model, the equation model showed *P* (0.0430) < 0.05, indicating that the regression model was significantly different, i.e., the model established was significant. As the fit term shows *P* (0.6011) > 0.05, it indicates that the model fits well with the actual experiment in the regression region. In addition, since the non-normal error in the actual experiment is small, the model values can be used instead of the actual experimental values for the analysis of the results. The coefficient of determination of the model R^2 = 0.8340 and the corrected coefficient of determination R^2_{Adj} = 0.6206, indicating that the model fits well and that the experimental method is reliable for the prediction and analysis of the honey-processing. The coefficient of variation CV = 43.48%, indicating good experimental stability. The above shows that the effect of each level factor on the response values is not purely linear. Three-dimensional response surface plots show a visualization of the interactions between the response of each variable and the experimental factors, as in Figure 1. Based on the results of the experiment, the optimum HRE processing parameters were: processing time 12.13 min, processing temperature 98.69 °C, and infiltration time 7.64 h; the OD value obtained was 0.756776. In order to facilitate practical operation, the processing time of 12 min, processing temperature of 100 °C, and infiltration time of 7.5 h were selected for experimental validation, and the obtained OD value was 0.800471, which was higher than the OD value predicted by the model, indicating that

the equation was well fitted and the extraction process obtained by response surface optimization was stable and feasible.

Table S1. Regression equations and linear ranges of three compounds.

Compound	Regression Equation	r^2	Linear Ranges/mg·L^{-1}
flavonoids	y=0.0052x-0.0017	0.9991	1.24–14.88
phenolic acids	y=0.0708x-0.0207	0.9994	4.56–54.72
alkaloids	y=0.0091x+0.3122	0.9994	20.00–80.00

Table S2. Response surface experimental design factor and level code.

Level	Processing Time (A) / min	Processing Temperature (B) / °C	Infiltration Time (C) / Hour
-1	10	90	5
0	12	100	7
1	14	110	9

Table S3. Responses for the Box–Behnken experimental design.

Run	A (min)	B (°C)	C (h)	Y
1	12	110	5	0.403005
2	10	100	9	0.537552
3	10	90	7	0
4	10	100	5	0.100377
5	12	100	7	0.5681
6	12	100	7	0.912048
7	12	100	7	0.512822
8	12	90	9	0.546291
9	14	90	7	0.309757
10	14	100	5	0.272025
11	12	100	7	0.818789
12	12	90	5	0.434546
13	14	110	7	0.147755
14	12	100	7	0.907462
15	12	110	9	0.218562
16	10	110	7	0
17	14	100	9	0.251814

Table S4. ANOVA for response surface models.

Term	Sum of Squares	Degrees of Freedom	Mean Square	*F*-value	*p*-value	Significance
Model	1.11	9	0.12	3.91	0.0430[c]	significant
Processing time, A	0.015	1	0.015	0.47	0.5160[d]	
Processing temperature, B	0.034	1	0.034	1.08	0.3337[d]	
Weight of medicinal herbs, C	0.015	1	0.015	0.47	0.5150[d]	
AB	6.561E-003	1	6.561E-00.	0.21	0.6620[d]	
AC	0.052	1	0.052	1.66	0.2386[d]	
BC	0.022	1	0.022	0.70	0.4317[d]	
A^2	0.58	1	0.58	18.27	0.0037[b]	
B^2	0.28	1	0.28	9.01	0.0199[c]	
C^2	0.029	1	0.029	0.93	0.3661[d]	
Residual	0.22	7	0.032			
Lack of fit	0.076	3	0.025	0.70	0.6011	not significant
Pure error	0.14	4	0.036			
Correlation total	1.33	16				
R^2	0.8340					
Adj. R^2	0.6206					

Level of significant expressed as $^{a}p < 0.001$; $^{b}p < 0.01$; $^{c}p < 0.05$; $^{d}p > 0.05$.

Figure S1. Structures of 65 compounds in P1 identified by HPLC-Q-TOF-MS.

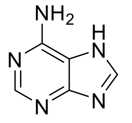
Adenine (1)

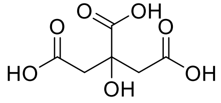
Citric acid (2)

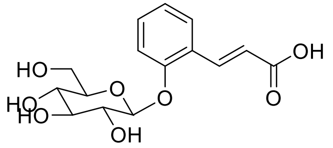
o-coumaric acid glucoside (3)

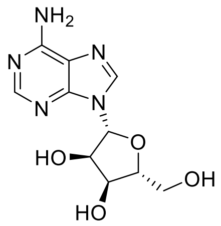
Adenosine (4)

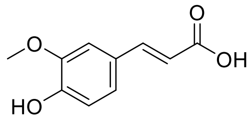
Ferulic Acid (5)

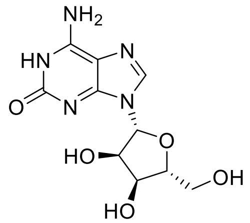
isoguanosine (6)

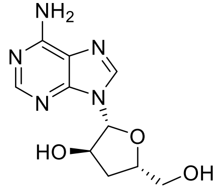
cordycepin (7)

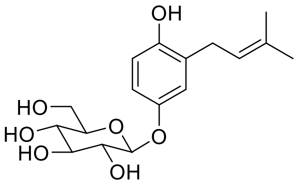
Nebrodenside A (8)

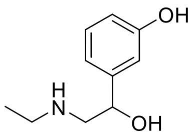
Etilefrine (9)

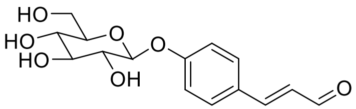
4-β-D-glucopyranosyloxy-trans-cinnamaldehyde (10)

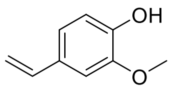
4-Vinylguaiacol (11)

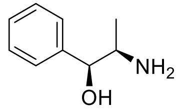
Norephedrine (12)

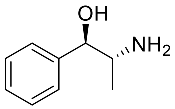
L-norpseudoephedrine (13)

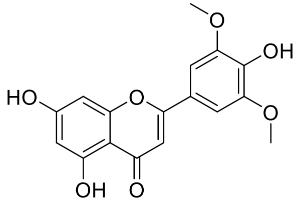
Tricin (14)

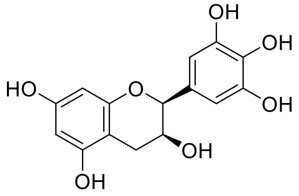
Gallocatechin (15)

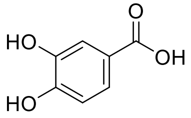
Protocatechuic acid (16)

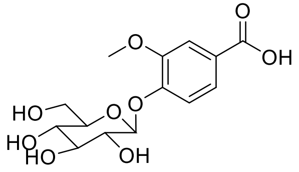
Pseudolaroside B (17)

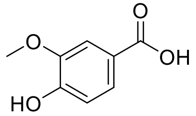
Vanillic acid (18)

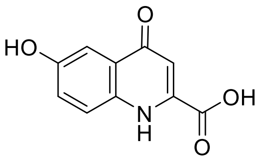

6-hydroxykynurenic acid (19)

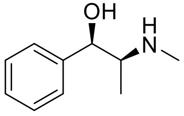

L-Ephedrine

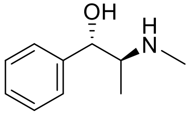

Pseudoephedrine (21)

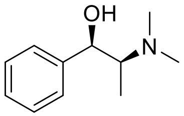

Methylephedrine (22)

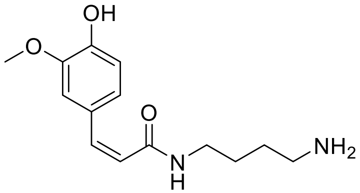

cis-N-feruloylputrescine (23)

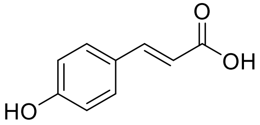

ρ-coumaric acid (24)

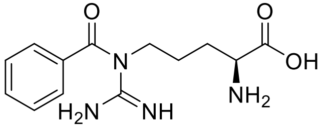

N''-benzoyl-l-arginine (25)

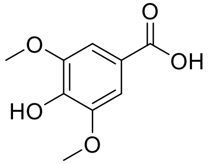

Syringic acid (26)

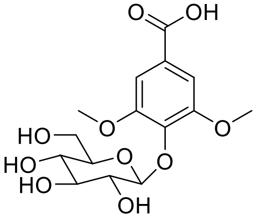

glucosyringic acid (27)

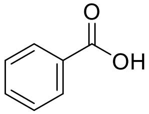

Benzoic acid (28)

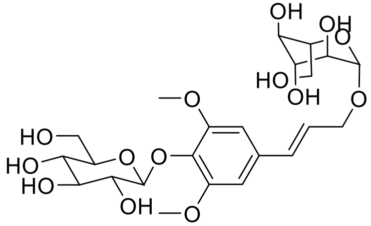

Isosyringinoside (29)

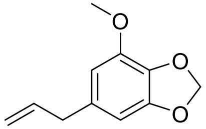

Myristicin (30)

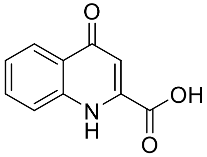

Kynurenic acid (31)

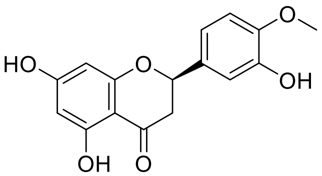

Hesperetin (32)

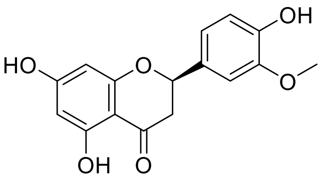

Homoeriodictyol (32)

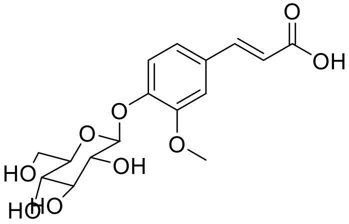

ferulic acid 4-O-β-D-glucopyranoside (33)

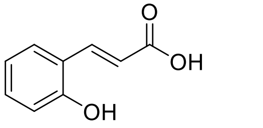

o-coumaric acid (34)

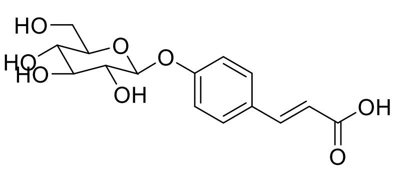
ρ-coumaric acid glucoside (35)

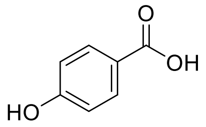
4-hydroxy-benzoic acid (36)

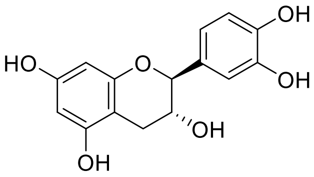
Catechin (37)

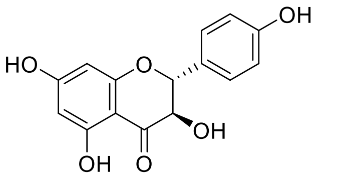
Aromadendrin (38)

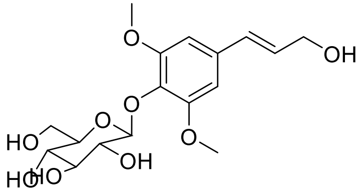
syringin (39)

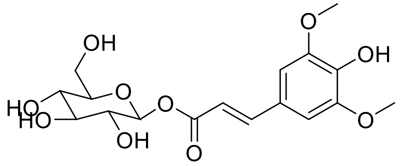
1-O-β-D-glucopyranosyl sinapate (40)

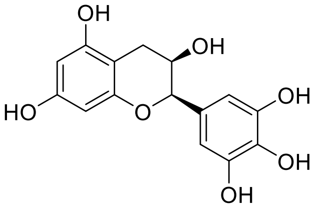
Epigallocatechin (41)

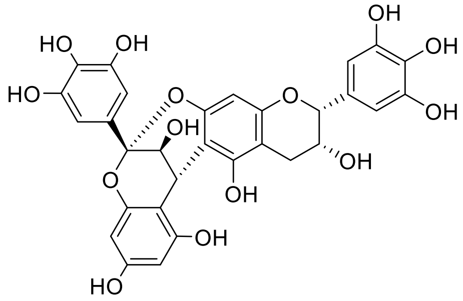
Gallocatechin- (4 → 6″; 2 → O → 7″)-(epi)gallocatechin (42)

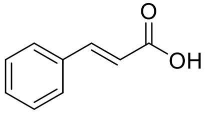
Cinnamic acid (43)

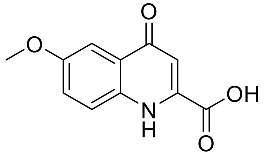
6-methoxykynurenic acid (44)

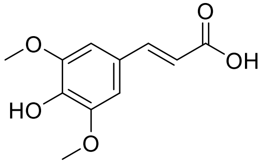
Sinapinic acid (45)

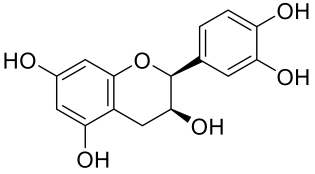
Epicatechin (46)

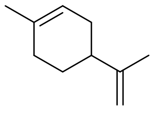
Limonene (47)

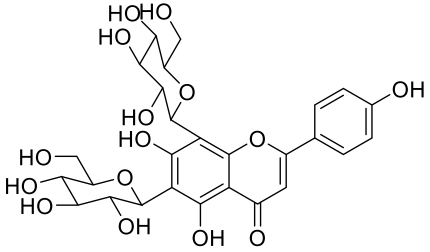
Vicenin-2 (48)

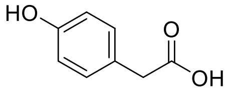

4-Hydroxyphenylacetic acid (49)

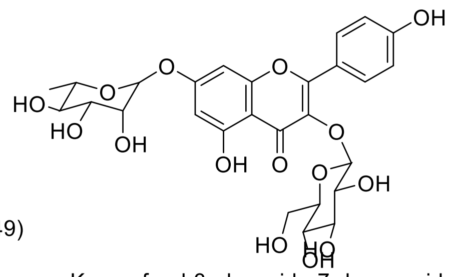

Kaempferol-3-glucoside-7-rhamnoside (50)

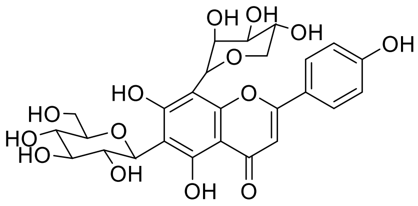

Schaftoside (51)

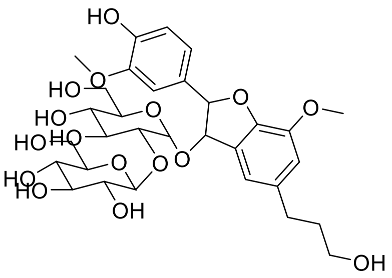

3-[2-(4-Hydroxy-3-methoxyphenyl)-3-[[2-O-(beta-D-glucopyranosyl)-alpha-D-glucopyranosyloxy]methyl]-7-methoxy-2,3-dihydrobenzofuran-5-yl]-1-propanol (52)

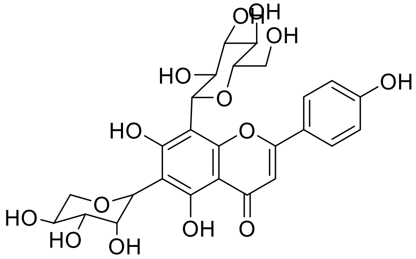

isoschaftoside (53)

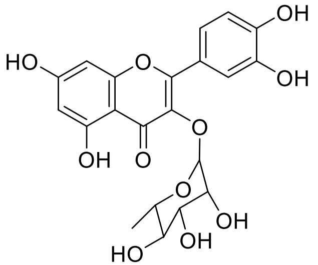

Quercetin-3-rhamnoside (54)

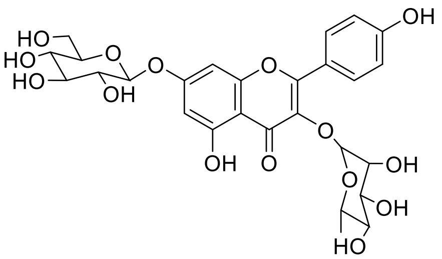

kaempferol-3-O-rhamnoside 7-O-glucoside (55)

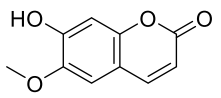

Scopoletin (56)

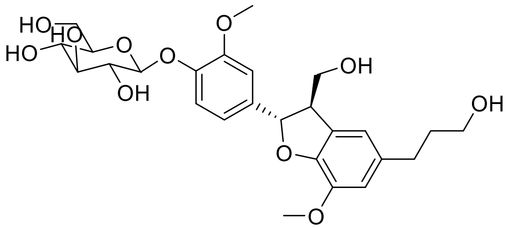

Urolignoside (57)

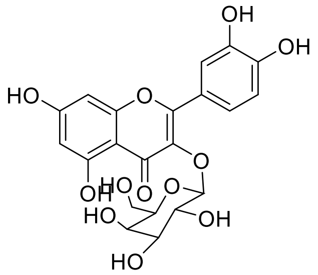

Hyperoside (58)

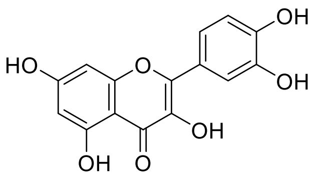

Quercetin (59)

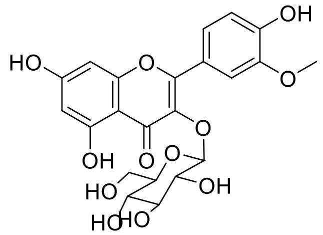

isorhamnetin-3-O-glucoside (60)

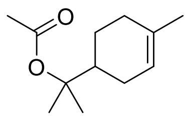

Terpineol acetate (61)

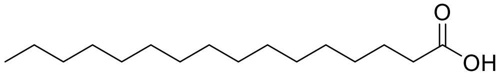

Hexadecanoic acid (62)

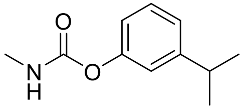

m-Cumenyl methylcarbamate (63)

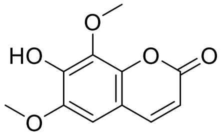

isofraxidin (64)

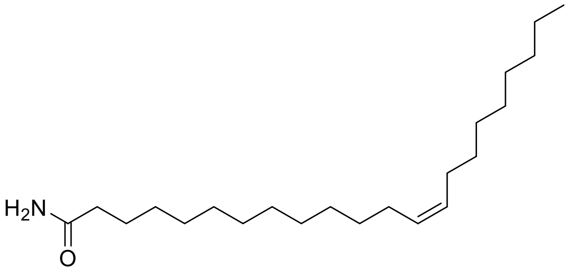

Erucylamide (65)

Figure S2. Structures of 38 compounds in P2 identified by HPLC-Q-TOF-MS.

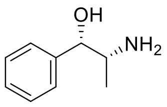

Norephedrine (1)

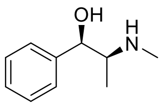

L-Ephedrine (2)

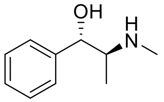

Pseudoephedrine (2)

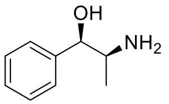

Methylephedrine (3)

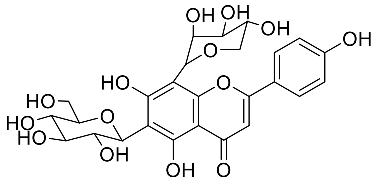

schaftoside (4)

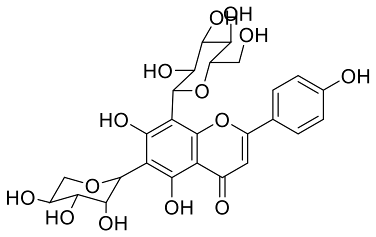

isoschaftoside (4)

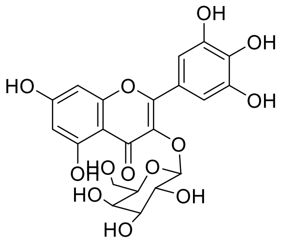

Myricetin-3-Galactoside (5)

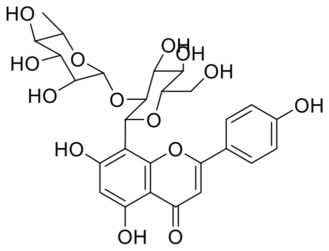

Vitexin-2-rhamnoside (6)

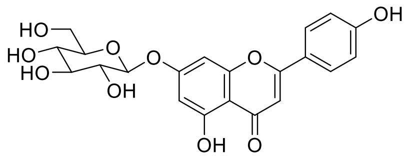

Apigenin-7-O-glucoside (7)

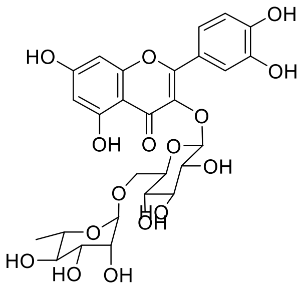

Rutin (8)

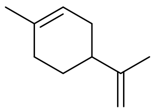

Limonene (9)

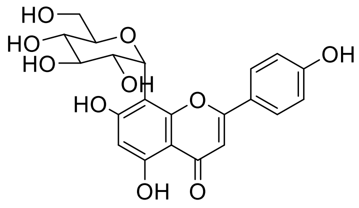

Vitexin (10)

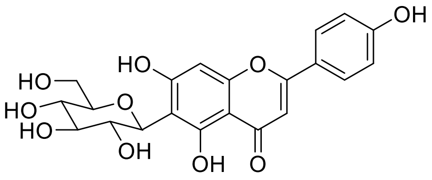

Isovitexin (10)

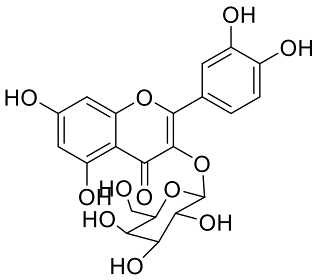

Hyperoside (11)

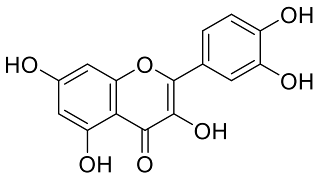

Quercetin (12)

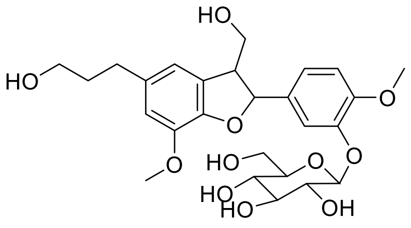

2-(Hydroxymethyl)-6-[5-[3-(hydroxymethyl)-5-(3-hydroxypropyl)-7-methoxy-2,3-dihydro-1-benzofuran-2-yl]-2-methoxyphenoxy]oxane-3,4,5-triol (13)

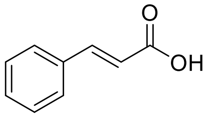

(Trans)-cinnamic acid (14)

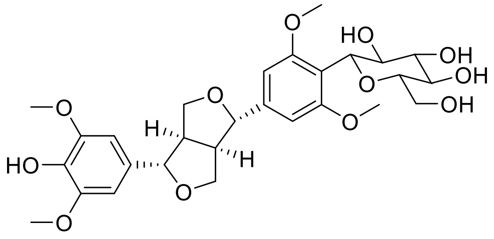

Acanthoside B (15)

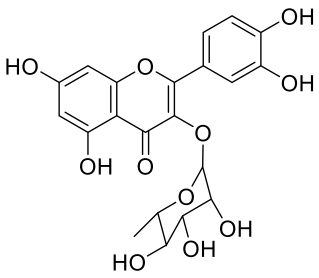

Quercetin-3-rhamnoside (16)

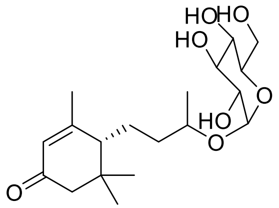

byzantionoside B (17)

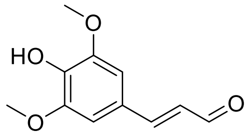

Sinapaldehyde (18)

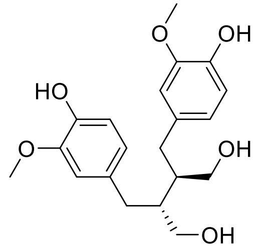

Secoisolariciresinol (19)

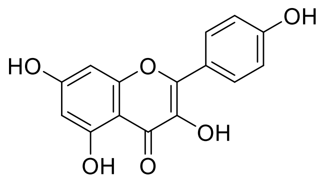

Kaempferol (20)

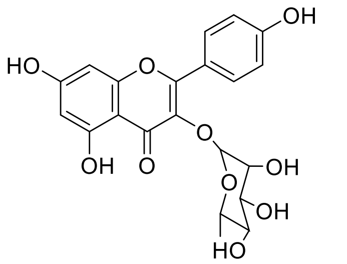

kaempferol-3-O-rhamnoside (21)

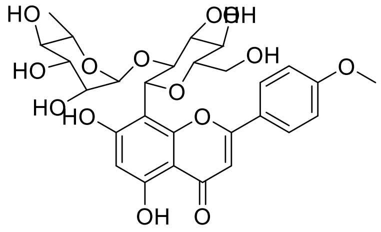

margaritene (22)

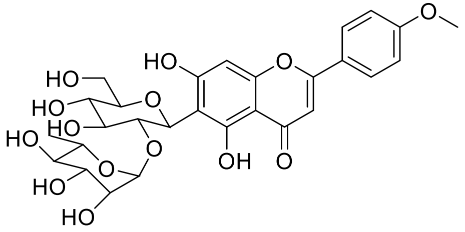

isomargaritene (22)

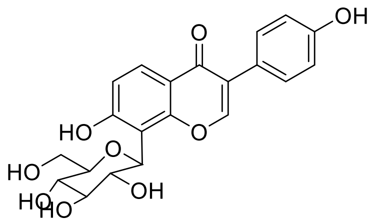

Puerarin (23)

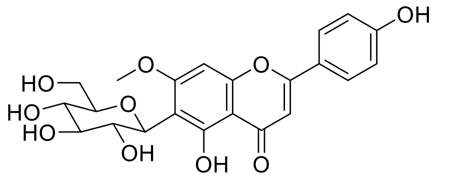

Swertisin (24)

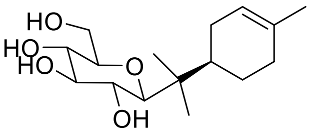

(−)-α-terpineol-8-O-β-D-glucopyranoside (25)

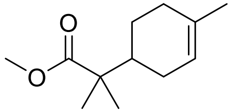

Terpineol acetate (26)

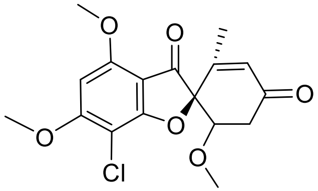

Griseofulvin (27)

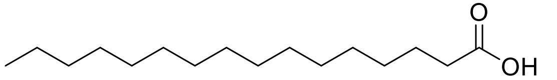

Hexadecanoic acid (28)

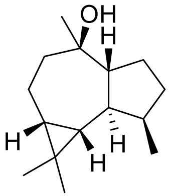

Globulol (29)

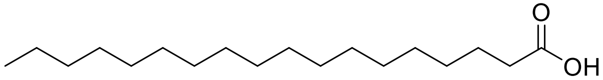

Stearic acid (31)

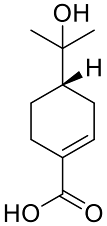

Oleuropeic acid (33)

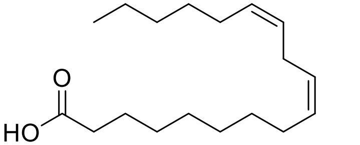

9,12-Linoleic acid (34)

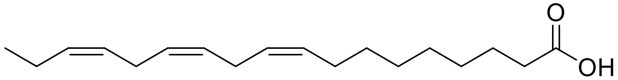

linolenic acid (32)

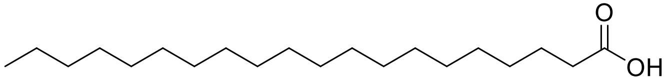

Eicosanoic acid (36)

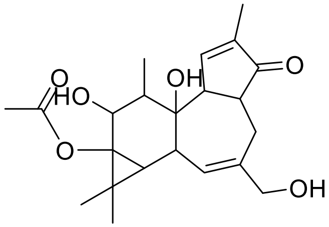

7b,9-Dihydroxy-3-(hydroxymethyl)-1,1,6,8-tetramethyl-5-oxo-1,1a,1b,4,4a,5,7a,7b,8,9-decahydro-9aH-cyclopropa[3,4]benzo[1,2-e]azulen-9a-yl acetate (30)

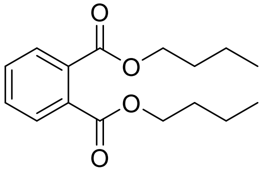

Dibutyl phthalate (35)

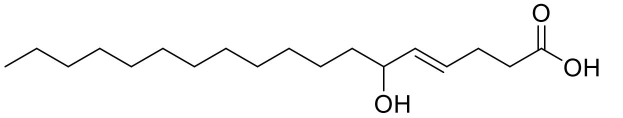

(E)-6-hydroxyoctadec-4-enoic acid (37)

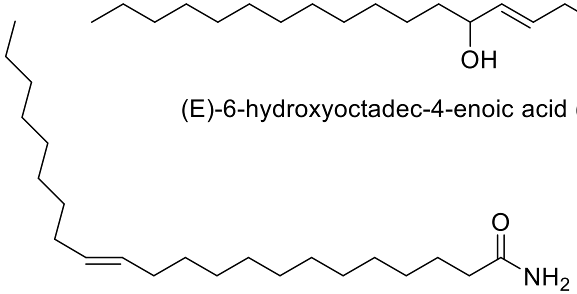

Erucylamide (38)

Figure S3. 3D view of response surface methodology of honey-processing Ehphedrae herba. (**A**) mutual effects of processing time and processing temperature on OD value; (**B**) mutual effects of processing time and infiltration time on OD value; (**C**) mutual effects of processing temperature and infiltration time on OD value.

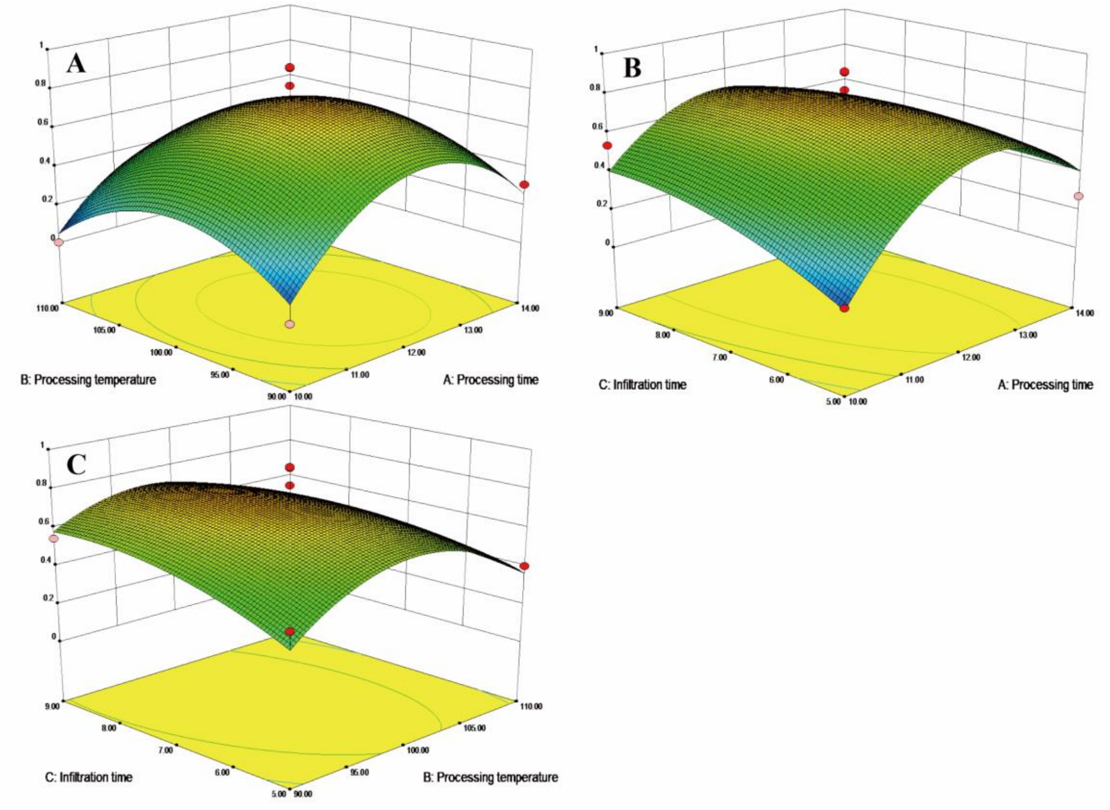

Supplement: Supplementary file 1 [file molecules-27-04057-s001.zip › molecules-1768131-supplementary.pdf]
